# The Association Between the Nursing Work Environment and Missed Nursing Care: A Systematic Review and Meta-Analysis

**DOI:** 10.3390/nursrep16090336

**Published:** 2026-09-15

**Authors:** Marin Mamić, Nikolina Farčić, Ivana Barać, Željko Mudri, Marija Barišić, Ivana Mamić, Zrinka Puharić, Gabriela Katharina Pomper, Ivan Vukoja

**Affiliations:** 1 General County Hospital Požega, Osječka 107, 34000 Požega, Croatia; ivana.mamic@pozeska-bolnica.hr (I.M.); gabrielakatharina.pomper@pozeska-bolnica.hr (G.K.P.); ivan.vukoja@pozeska-bolnica.hr (I.V.); 2 Faculty of Dental Medicine and Health Osijek, Josip Juraj Strossmayer University of Osijek, Crkvena 21, 31000 Osijek, Croatia; nfarcic@fdmz.hr (N.F.); ibarac@fdmz.hr (I.B.); zmudri@fdmz.hr (Ž.M.); mbarisic@fdmz.hr (M.B.); zpuharic@vub.hr (Z.P.); 3 Department of Neurosurgery, University Hospital Centre Osijek, Josip Huttler Street 4, 31000 Osijek, Croatia; 4 Department of Nursing, Bjelovar University of Applied Sciences, Trg Eugena Kvaternika 4, 43000 Bjelovar, Croatia; 5 Faculty of Medicine, Josip Juraj Strossmayer University of Osijek, J. Huttlera 4, 31000 Osijek, Croatia; 6 Faculty of Medicine, University of Rijeka, Braće Branchetta 20, 51000 Rijeka, Croatia

**Keywords:** missed care, incomplete care, nursing work environment, nursing practice environment, PES-NWI, systematic review, meta-analysis

## Abstract

**Background/Objectives**: Missed nursing care threatens care quality and patient safety and may reflect modifiable organizational conditions. This systematic review and meta-analysis quantified the association between the nursing work environment and missed nursing care. **Methods**: PubMed, Scopus, Web of Science, and APA PsycInfo via EBSCOhost were searched from inception; searches were updated and finalized on 13 July 2026. Quantitative studies examining nursing work, professional, or practice environments in relation to missed, incomplete, unfinished, omitted, or implicitly rationed nursing care were eligible. The eligibility criteria, primary total-score Pearson correlation analysis, random-effects model with restricted maximum likelihood (REML) estimation, and direction harmonization were specified before inspection of the pooled results. Additional robustness and exploratory analyses were conducted post hoc, including Hartung-Knapp adjustment, leave-one-out and influence analyses, risk-of-bias and instrument-family sensitivity analyses, alternative correlation-metric analyses, meta-regression, and small-study-effect assessments. The review was retrospectively registered in the Open Science Framework on 25 August 2026 (DOI: 10.17605/OSF.IO/J62D3); no prospective protocol was available. **Results**: Forty studies were included. The primary meta-analysis comprised 15 studies and 7100 participants. A more favorable nursing work environment was associated on average with less missed nursing care (*r* = −0.301; 95% CI −0.402 to −0.192; *p* < 0.001; model-based *I*^2^ = 95.7%). The 95% prediction interval ranged from −0.641 to 0.139 and crossed the null, indicating substantial uncertainty about the magnitude and potentially the direction of the association in a new setting. The association remained statistically significant with Hartung-Knapp adjustment (95% CI −0.411 to −0.182; *p* < 0.001), and no leave-one-out model changed its direction or statistical significance. **Conclusions**: On average, a more supportive nursing work environment was associated with less missed nursing care. However, the very high heterogeneity and a prediction interval crossing the null indicate substantial variation across settings. The pooled coefficient therefore represents an average association rather than a universal effect, and the predominantly observational evidence does not establish that modifying the work environment will causally reduce missed nursing care.

## 1. Introduction

Missed nursing care refers to necessary patient care that is partially or completely omitted or significantly delayed [1,2]. Related terms include unfinished nursing care, care left undone, omitted nursing care, implicit rationing of nursing care, and nursing care omissions [2,3]. These labels are not strictly interchangeable: they arose from partly different conceptual traditions, may emphasize different mechanisms (for example, omission, incompletion, or implicit rationing), and are operationalized with different instruments and recall frames. They were nevertheless considered eligible within the same review because each captures the broader process-level phenomenon of required nursing care not being completed at the expected time or to the required extent. This conceptual commonality justified the broad systematic-review scope, whereas quantitative comparability was addressed by restricting the primary meta-analysis to total-score Pearson correlations and by conducting post hoc instrument-family sensitivity analyses.

Missed nursing care is important because it is an indicator of the health care process and thus the quality and safety of care. Previous research has linked the omission of necessary care with worse perceived quality of care, lower patient satisfaction, adverse treatment outcomes, nurse burnout, job dissatisfaction and intention to leave the profession [3,4,5]. Commonly missed activities include patient mobilization and repositioning, oral hygiene and care, patient and family education, emotional support, discharge planning, development of care plans, and participation in interdisciplinary meetings [1,3,4,5].

The occurrence of missed care should not be interpreted solely as a consequence of individual decisions or professional failures of the individual nurse. Care is provided within organizational systems that determine the number and structure of staff, availability of material resources, workload, professional autonomy, managerial support, participation in decision-making, interdisciplinary relationships, and a broader culture of quality and patient safety. When demands for care exceed available organizational capacities, nurses may be forced to prioritize certain activities and postpone or omit others [2,3].

The nursing work environment is the organizational context in which nurses provide nursing care. Lake developed the Practice Environment Scale of the Nursing Work Index (PES-NWI), one of the most commonly used instruments to assess this construct, which includes nurses’ participation in hospital affairs, nursing foundations for quality of care, nurse manager ability, leadership and support, staffing and resource adequacy, and collegial nurse–physician relations [6]. More favorable nursing work environments are associated with better professional outcomes for nurses, better quality of care, greater patient safety, and fewer adverse outcomes [6,7]. The association between the nursing work environment and missed care can also be interpreted within Donabedian’s structure, process, and outcome model [8]. The nursing work environment represents a structural characteristic of the care delivery system because it includes the organizational conditions in which care is provided. Missed care represents a process indicator because it reflects the extent to which required activities are actually performed in a timely manner and to the required extent. Accordingly, unfavorable structural conditions can be reflected in disruptions in the care process and an increased frequency of omitted or delayed activities.

Previous systematic and review work has identified associations between the nursing work environment and missed or incomplete nursing care [9,10,11]. In particular, Zhao et al. systematically reviewed the association between work environment and implicit rationing of nursing care but synthesized the findings narratively and searched the literature only through May 2019 [11]. Other reviews have examined broader organizational factors or multiple professional and patient outcomes [9,10]. The present review adds a quantitative synthesis centered on a deliberately strict primary effect definition: the total-score Pearson correlation between the overall nursing work environment and overall missed nursing care. It also separates different effect-size families, reports prediction intervals and influence diagnostics, and examines robustness to risk-of-bias and measurement-instrument restrictions. This approach is intended to estimate the average cross-study association while making the substantial conceptual and statistical heterogeneity explicit.

Therefore, the goal of this systematic review and meta-analysis was to quantify the association between the nursing work environment and missed nursing care using a prespecified primary total-score Pearson correlation model. Post hoc analyses were then used to examine robustness, influential studies, measurement-related heterogeneity, risk-of-bias restrictions, possible moderators, and small-study effects, while additional studies reporting odds ratios, regression coefficients, longitudinal effects, or other measures of association were synthesized separately or narratively.

## 2. Materials and Methods

### 2.1. Research Design and Reporting Guidelines

The systematic review and meta-analysis were conducted in accordance with the Preferred Reporting Items for Systematic Reviews and Meta-Analyses 2020 (PRISMA 2020) guidelines [12]. Search reporting was additionally structured with reference to PRISMA-S [13].

The review and meta-analysis were retrospectively registered in the Open Science Framework (OSF) on 25 August 2026 (DOI: 10.17605/OSF.IO/J62D3), after study screening, data extraction, and the primary statistical analyses had been completed. No publicly available prospective protocol existed before the review procedures began. Before inspection of the pooled results, the authors had specified the eligibility criteria, the primary meta-analysis of total-score Pearson correlations, the use of a random-effects model with REML estimation, and the harmonization of effect direction. Hartung-Knapp, leave-one-out, exclusion of the directionally discrepant Amiri Amjad et al. study, Spearman/Kendall sensitivity analyses, risk-of-bias and instrument-family sensitivity analyses, meta-regressions, influence diagnostics, Egger and rank-correlation tests, and trim-and-fill were subsequently added as post hoc robustness or exploratory analyses. This distinction is reported to minimize ambiguity about confirmatory versus data-informed analyses.

### 2.2. Research Question

The research question was formulated according to the PECO framework.

The population consisted of nurses who directly participate in the provision of nursing care.

Exposure was the nursing practice, work, or professional environment as assessed by a multidimensional organizational instrument.

The comparison, where applicable, was between more favorable and less favorable work environments or exposure was analyzed as a continuous variable.

The outcome included missed, incomplete, unperformed, omitted, or implicitly rationed nursing care.

The primary research question was:

What is the association between the nursing work environment and missed nursing care among direct patient care nurses?

### 2.3. Information Sources and Search Strategies

PubMed, Scopus, Web of Science, and APA PsycInfo via the EBSCOhost platform were systematically searched from inception, with the initial search and screening process conducted between 15 March and 15 June 2026. All database searches were subsequently updated and finalized on 13 July 2026 to identify newly published or indexed records before completion of the review. CINAHL was not available through the authors’ institutional access and therefore was not searched. To broaden retrieval, database searching was supplemented by backward reference-list screening, forward citation tracking, Google Scholar and publisher-site searches, and targeted exact-title and concept searches. These supplementary approaches increased coverage but were not considered a complete substitute for CINAHL; omission of CINAHL remains an important limitation because nursing-specific records may have been missed. No publication-year restriction was applied.

The search strategy for PubMed was:

(

“nursing practice environment” [Title/Abstract]

OR “nurse practice environment” [Title/Abstract]

OR “professional practice environment” [Title/Abstract]

OR “nursing work environment” [Title/Abstract]

OR “practice environment scale” [Title/Abstract]

OR “PES-NWI” [Title/Abstract]

)

AND

(

“missed nursing care” [Title/Abstract]

OR “unfinished nursing care” [Title/Abstract]

OR “care left undone” [Title/Abstract]

OR “implicit rationing of nursing care” [Title/Abstract]

OR “rationing of nursing care” [Title/Abstract]

OR “omitted nursing care” [Title/Abstract]

OR “nursing care omissions” [Title/Abstract]

)

AND

(

nurse [Title/Abstract]

OR nurses [Title/Abstract]

OR nursing [Title/Abstract]

)

The strategies were adapted to the syntax of Scopus, Web of Science, and APA PsycInfo via EBSCOhost. The exact EBSCOhost database, search dates, complete database strategies, fields, limits, and documented supplementary-search procedures are reported in Supplementary Table S1.

### 2.4. Inclusion and Exclusion Criteria

The following eligibility criteria were specified before inspection of the pooled results. Eligible studies were quantitative cross-sectional, longitudinal, or other observational studies that included direct-care nurses; assessed the nursing work, practice, or professional environment as a multidimensional organizational construct; assessed missed, incomplete, unfinished, omitted, implicitly rationed, or otherwise unperformed nursing care; and reported an association between the two constructs, irrespective of direction or statistical significance.

The primary quantitative synthesis was also specified in advance. Studies entered the primary meta-analysis only when they reported a Pearson correlation coefficient between a total score for the nursing work/practice environment and a total score for missed nursing care. Direction harmonization was prespecified so that a negative analysis-ready coefficient consistently represented a more favorable work environment associated with a lower level of missed care. The instrument used in each study, its scoring orientation, and any sign reversal are reported at the study level in Supplementary Table S10.

Analyses involving Spearman coefficients, the approximate conversion of Kendall’s τ, and the separate pooling of adjusted odds ratios were not part of the prespecified primary model. They were added post hoc as sensitivity or exploratory analyses and were kept separate from the primary Pearson synthesis because they represent different association metrics or operationalizations.

Studies that reported only associations of individual work environment subscales with individual missed care activities were included in the systematic review and narrative synthesis but not in the primary total–total correlation meta-analysis.

Studies with mixed work groups were considered for the narrative synthesis when they directly addressed the research question but were not included in the primary meta-analysis if results for the target population of nurses were not separately available.

Conference abstracts and reports without sufficient methodological and numerical data were not included in the main quantitative synthesis. When a preprint and a later published peer-reviewed version presented the same research and the same sample, they were treated as a single study, with the final peer-reviewed publication taking precedence.

Eligibility for the systematic review and eligibility for the primary meta-analysis were therefore treated as two distinct levels. Studies meeting the systematic-review eligibility criteria remained included in the systematic review and narrative synthesis even when they did not meet the more restrictive eligibility criteria for the primary Pearson correlation meta-analysis. The detailed eligibility criteria and screening stages are summarized in Table 1.

### 2.5. Screening and Selection Procedures

Two reviewers (M.M. and I.M.) independently screened titles and abstracts and subsequently assessed potentially eligible full-text reports. Disagreements were resolved through discussion and consensus. Reasons for exclusion at the full-text stage were recorded in the screening file. Retrieved records were first imported into Zotero (version 9.0.4), where duplicates were identified and removed. Additional duplicate checking was then performed in the screening workbook using DOI, PMID, and title, followed by manual comparison of near-duplicate records based on similar titles, first authors, publication years, journals, and study samples.

A search of four bibliographic sources identified 292 records: 44 in PubMed, 55 in Scopus, 145 in Web of Science and 48 in APA PsycInfo via EBSCOhost. After removing 205 duplicates, 87 unique records remained for title and abstract review.

At title and abstract screening, 47 records were excluded, and 40 reports were sought for retrieval. Despite searches through institutional subscriptions, publisher websites, Google Scholar, reference lists, and open-access repositories, one potentially relevant report could not be retrieved. Consequently, 39 reports identified through database searching were assessed in full text.

Supplementary searches were conducted during the same review period and finalized on 13 July 2026. Backward reference-list screening and forward citation tracking used the prior reviews by Chaboyer et al. and Zhao et al. [10,11] and all provisionally eligible reports as seed records. Google Scholar, PubMed-related/citing records, publisher sites, and reference lists were searched using the principal database concept combinations and exact-title searches. Seven additional unique reports remained after deduplication and were assessed in full text. Across all sources, 46 full-text reports were assessed; six were excluded for documented reasons [14,15,16,17,18,19], leaving 40 unique studies in the systematic review.

Of these 40 studies, 15 were included in the primary Pearson correlation meta-analysis, three additional studies with Spearman coefficients were included in sensitivity analyses, one study with Kendall’s τ was included only in the exploratory sensitivity analysis, and three studies contributed to a separate exploratory meta-analysis of odds ratios. The remaining studies contributed to the narrative synthesis. Some synthesis categories overlapped, but each unique study was counted only once in the total number of included studies.

Accordingly, 25 studies met the eligibility criteria for inclusion in the systematic review but did not contribute to the prespecified primary Pearson correlation meta-analysis.

**Figure 1:**
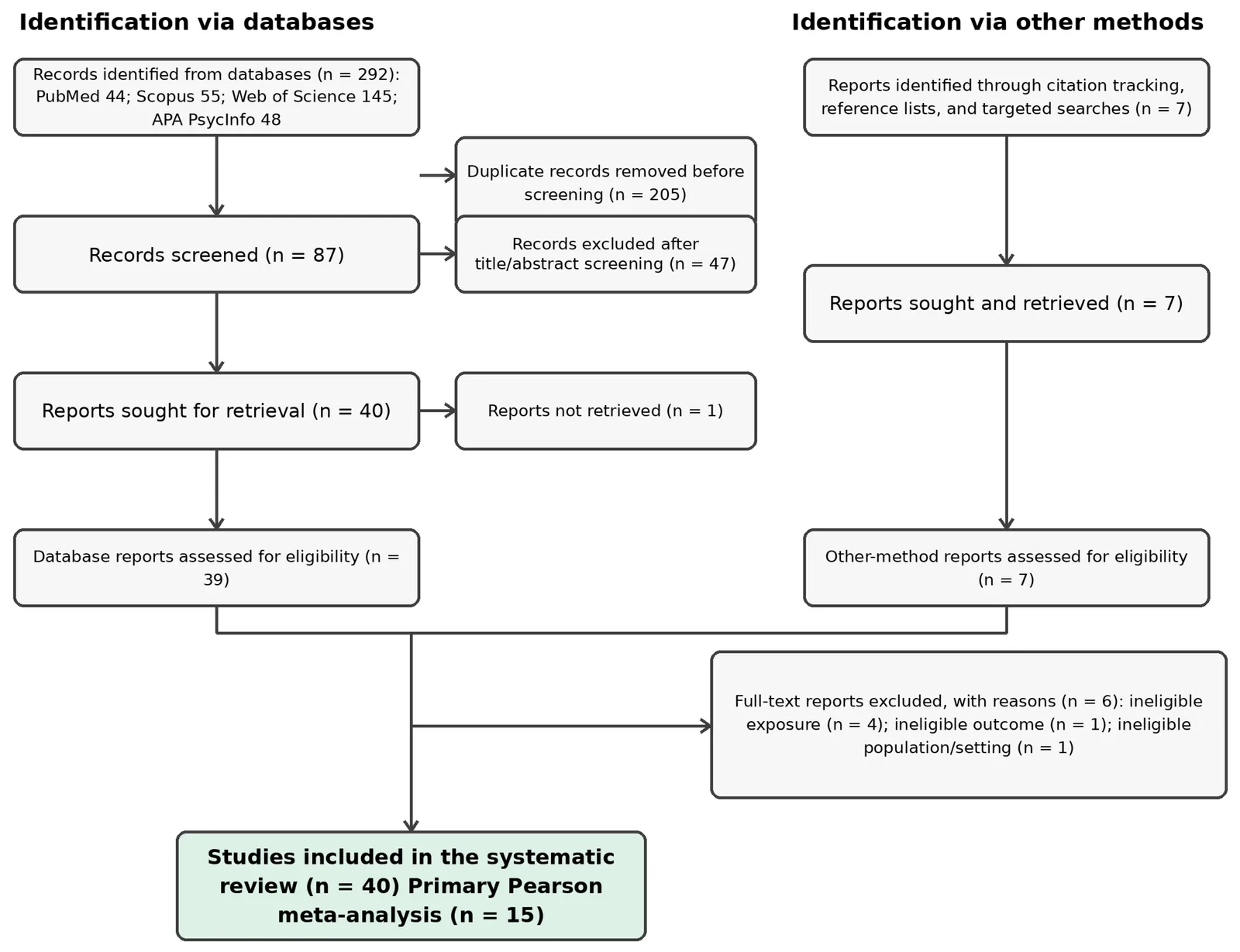
PRISMA 2020 flow diagram for identification, screening, eligibility assessment, and inclusion.

The PRISMA 2020 flow diagram is shown in Figure 1. Full-text exclusion reasons and the report not retrieved are listed in Supplementary Tables S5 and S6, respectively [14,15,16,17,18,19,20].

### 2.6. Data Extraction

Data extraction was conducted from 20 May to 15 July 2026. For each included study, the author and year of publication, country, study design, clinical setting, sample size, participant characteristics, nursing work-environment assessment instrument, missed-care assessment instrument, type of statistical analysis, effect measure, scoring direction, and, where applicable, variables included in adjusted models were extracted using a standardized extraction framework. M.M. performed the extraction, and I.M. independently verified all extracted variables for all included studies against the full reports. Any discrepancies were resolved through discussion and re-checking of the source publication. The 40 included reports are listed in Supplementary Table S3 [21,22,23,24,25,26,27,28,29,30,31,32,33,34,35,36,37,38,39,40,41,42,43,44,45,46,47,48,49,50,51,52,53,54,55,56,57,58,59,60].

For the primary meta-analysis, Pearson correlation coefficients and associated sample sizes were extracted. For sensitivity analyses, Spearman coefficients were recorded. For a separate meta-analysis of odds ratios, ORs, adjusted ORs, and 95% confidence intervals were extracted.

When instrument scoring direction differed across studies, effects were harmonized according to the prespecified convention that negative values indicate a more favorable work environment associated with less missed care. The sign was reversed only when the original scoring orientation required it; the coefficient magnitude was not changed. Menard [31] required sign reversal because the study-specific scoring of the two constructs produced an effect in the opposite algebraic direction from the review convention. The positive coefficient reported by Amiri Amjad et al. [43] was retained as published and was not reversed. Study-level scoring direction and harmonization decisions are documented in Supplementary Table S10.

### 2.7. Risk of Bias Assessment

Risk of bias was assessed according to study design using the revised JBI tools for analytical cross-sectional and cohort studies [61,62].

For cross-sectional studies, the revised eight-item JBI analytical cross-sectional tool was used. Its items assess the clarity of inclusion criteria; description of study participants and setting; validity and reliability of exposure measurement; use of objective, standard criteria for measurement of the condition; identification and management of confounding; validity and reliability of outcome measurement; and appropriateness of statistical analysis. The item concerning objective, standard criteria for measuring the condition (Question 4) was treated as not applicable when participant inclusion did not depend on an objectively diagnosed condition. For longitudinal studies, the revised 11-item JBI cohort tool was used.

No total numerical JBI score was calculated, nor was an arbitrary threshold applied to categorize studies as low, moderate, or high quality. Assessments were kept at the individual item level.

Risk of bias was independently assessed by two reviewers (M.M. and N.F.) using the study-design-specific revised JBI tools. Disagreements were resolved through discussion and consensus. No study was excluded solely on the basis of its JBI appraisal. JBI judgments were interpreted at the domain level rather than converted to an arbitrary total quality score. Because risk of bias was not part of the weighting scheme of the prespecified primary model, two post hoc sensitivity analyses were added: one excluding studies with an explicit “No” in mapped key domains and a stricter analysis retaining only studies rated “Yes” in all mapped key domains relevant to exposure measurement, confounding, outcome measurement, and statistical analysis.

### 2.8. Effect Measures

The primary outcome measure was the Pearson correlation coefficient *r* between the nursing work environment total score and the missed nursing care total score.

Correlation coefficients were transformed by Fisher’s *z*-transformation:

*z* = 0.5 × ln[(1 + *r*)/(1 − *r*)] (1)

The variance was calculated according to the expression:

*V* = 1/(*N* − 3) (2)

and standard error:

*SE* = √[1/(*N* − 3)]. (3)

This conventional study-level sampling variance was used when a correlation and sample size were reported without a study-specific standard error.

After pooling, Fisher’s *z*-values and associated confidence intervals were back-transformed to Pearson’s *r* for interpretation.

Spearman coefficients were included only in sensitivity analyses. Kendall’s τ was considered in an additional exploratory analysis using an approximate transformation:

*r* ≈ sin(π*τ*/2). (4)

Odds ratios were log-transformed before pooling, and after analysis, the results were retransformed to OR for interpretation.

### 2.9. Statistical Analysis

The prespecified primary analysis used a random-effects model because clinical, methodological, organizational, and health-system differences among studies were expected. Between-study variance was estimated using restricted maximum likelihood (REML). The conventional *z*-based REML model was treated as the primary inferential model because it corresponded to the prespecified analysis; Hartung-Knapp was added post hoc as a robustness analysis to examine the sensitivity of interval estimation with only 15 primary studies.

Heterogeneity was assessed using Cochran’s *Q*, τ^2^, and the model-based *I*^2^ returned by metafor. *I*^2^ was interpreted as the proportion of total variability attributable to between-study heterogeneity under the fitted random-effects model. *Q*-profile 95% confidence intervals for τ^2^ and model-based *I*^2^ were calculated with confint(model, type = “QP”). A 95% prediction interval was also calculated for the primary model to show the expected range of true effects in comparable future settings.

The primary meta-analysis included only total-score Pearson correlations. After the primary results were inspected, post hoc robustness analyses were added: Hartung-Knapp adjustment; leave-one-out and influence diagnostics; exclusion of Amiri Amjad et al.; Pearson-plus-Spearman analyses; an exploratory Kendall-τ conversion analysis; overlap sensitivity for the two Zeleníková reports; risk-of-bias restrictions; and instrument-family restrictions. These additional analyses were not treated as confirmatory alternatives to the prespecified primary model.

Sensitivity analyses excluded the directionally different study of Amiri Amjad et al. [43], included three additional Spearman correlations, repeated the combined Pearson and Spearman analysis without Amiri Amjad et al., and included an approximately converted Kendall coefficient in an additional exploratory model. The primary REML model was also repeated using the Hartung–Knapp adjustment. Leave-one-out analysis sequentially omitted each primary study. Influence diagnostics included deleted studentized residuals, hat values, Cook’s distances, and DFBETAS. Values of |deleted studentized residual| > 2, Cook’s distance > 4/*k*, hat value > 2/*k*, and |DFBETAS| > 2/√*k* were treated as indicators warranting closer examination rather than automatic grounds for exclusion. Because partial sample overlap between Zeleníková et al. 2020 and 2021 [37,58] could not be excluded, the Pearson-plus-Spearman model was repeated after omitting each report separately.

For the three studies with sufficiently comparable odds ratios, a separate meta-analysis of ORs was performed.

Three post hoc univariable exploratory meta-regressions examined publication year, missed-care instrument family (MISSCARE family, *k* = 9; other, *k* = 6), and work-environment instrument family (PES/NWI family, *k* = 10; other, *k* = 5). Binary instrument classifications were used only as coarse exploratory indicators; the “other” categories contain conceptually heterogeneous instruments, so non-significant moderator tests cannot be interpreted as evidence of measurement equivalence.

Potential small-study effects were assessed post hoc using visual funnel-plot inspection, Egger’s regression test, the Begg-Mazumdar rank-correlation test, and Duval-Tweedie trim-and-fill as an additional sensitivity analysis. These procedures were interpreted as exploratory because only 15 studies were available and heterogeneity was very high; they assess funnel asymmetry or small-study effects rather than proving the presence or absence of publication bias.

Statistical analyses were conducted from 16 to 22 July 2026 in R version 4.5.2 (R Foundation for Statistical Computing, Vienna, Austria) [63] using the metafor package version 5.0-1 [64]. The complete reproducible script, analysis-ready workbook, exported numerical results, and session information are provided in the Supplementary Materials.

## 3. Results

### 3.1. Study Selection

The database searches identified 292 records: PubMed (*n* = 44), Scopus (*n* = 55), Web of Science (*n* = 145), and APA PsycInfo via EBSCOhost (*n* = 48). After 205 duplicates were removed, 87 unique records were screened and 47 were excluded on title and abstract. Forty reports were sought for retrieval; one could not be retrieved, and 39 database reports were assessed in full text. Seven additional reports identified through targeted supplementary searches were also assessed, resulting in 46 full-text reports assessed. Six reports were excluded after full-text assessment, leaving 40 studies in the systematic review; 15 contributed to the primary Pearson meta-analysis.

Fifteen studies were eligible for the primary Pearson correlation meta-analysis. Three additional studies reporting Spearman coefficients were included in sensitivity analyses, one study reporting Kendall’s τ was added only to an exploratory sensitivity analysis, and three studies contributed to a separate exploratory meta-analysis of odds ratios.

### 3.2. Characteristics of Studies Included in the Primary Pearson Meta-Analysis

The 40 included studies are presented in Supplementary Table S3 and correspond to references [21,22,23,24,25,26,27,28,29,30,31,32,33,34,35,36,37,38,39,40,41,42,43,44,45,46,47,48,49,50,51,52,53,54,55,56,57,58,59,60]. Detailed characteristics and extracted findings are provided in Supplementary Table S3.

Fifteen studies included in the primary Pearson meta-analysis were published between 2014 and 2026 and included a total of 7100 participants.

Fourteen of the 15 studies showed the theoretically expected inverse direction; the only directionally discrepant positive result was reported by Amiri Amjad et al. [43].

The strongest inverse associations were reported by Menard [31], Kim and Uhm [54], and Lu et al. [53], whereas Amiri Amjad et al. reported the only positive coefficient [43]. Study-level characteristics for the primary Pearson meta-analysis are summarized in Table 2.

Smith et al. reported a significant total–total association (*r* = −0.20) in 233 nurses [25]. Babaei et al. found *r* = −0.18 in 255 nurses; in their regression model, nursing foundations for quality of care was the only practice-environment dimension independently associated with missed care [29]. Reassessed reports by Zeleníková et al. [58], Caillet et al. [59], and Caponnetto et al. [60] contributed, respectively, to the Spearman sensitivity analysis or the narrative synthesis because the latter two did not report an eligible overall total–total coefficient. In Lu et al., *r* = −0.502 linked work environment measured at T1 with missed nursing care measured at T3 [53].

### 3.3. Assessment of Risk of Bias

Across the cross-sectional studies, inclusion criteria, exposure and outcome measurement, statistical analysis, and descriptions of participants and settings were generally appropriate in the available full reports. The most recurrent concern involved identification or management of confounding. Risk-of-bias judgments were not used to alter study weights in the prespecified primary model. In the post hoc analysis excluding studies with any explicit “No” in mapped key JBI domains, the pooled association remained similar (*k* = 14; *N* = 6717; *r* = −0.296; 95% CI −0.405 to −0.179; *I*^2^ = 96.0%). In the stricter analysis retaining only studies rated “Yes” in all mapped key domains, the association was *r* = −0.358 (*k* = 11; *N* = 6001; 95% CI −0.443 to −0.266; *I*^2^ = 93.2%), with a 95% prediction interval of −0.611 to −0.038. Thus, the average inverse association persisted under a stricter risk-of-bias restriction, although substantial heterogeneity remained.

These methodological limitations were taken into account when interpreting the results but were not automatically used as a reason for excluding the study.

The full assessment of each included study by individual JBI questions is shown in Supplementary Table S4A,B.

### 3.4. A Primary Meta-Analysis of Pearson Correlations

The pooled association between the nursing work environment and missed nursing care was *r* = −0.301 (95% CI −0.402 to −0.192; *p* < 0.001). The confidence interval describes uncertainty around the estimated mean association across the included studies and did not include zero. In contrast, the 95% prediction interval, which incorporates estimated between-study heterogeneity and describes the range in which a true effect from a new comparable setting could plausibly fall, ranged from −0.641 to 0.139 and crossed zero. This distinction is central to interpretation: the average association was statistically different from zero, but an association near zero or in the opposite direction remains plausible in some new settings.

Very high heterogeneity was found, *Q*(14) = 361.599; *p* < 0.001; model-based *I*^2^ = 95.7% (*Q*-profile 95% CI 91.8% to 98.2%); τ^2^ = 0.049 (*Q*-profile 95% CI 0.025 to 0.125). The 95% prediction interval, from *r* = −0.641 to *r* = 0.139, crossed the null, indicating that although the average association was inverse, a negligible or directionally different true association remains plausible in some comparable settings. The primary model results are summarized in Table 3.

### 3.5. Sensitivity Analyses

Because Amiri Amjad et al. [43] was the only study in the primary model reporting a positive association, a sensitivity analysis was performed after its exclusion. The remaining 14 studies included 6720 participants. The magnitude of the association strengthened to *r* = −0.340 (95% CI −0.413 to −0.263; *p* < 0.001), while heterogeneity remained high (model-based *I*^2^ = 91.3%; τ^2^ = 0.023). The prediction interval ranged from *r* = −0.581 to *r* = −0.045 and did not include the null.

The REML model with Hartung–Knapp adjustment produced the same pooled estimate, *r* = −0.301, with a 95% CI from −0.411 to −0.182, *t*(14) = −5.273; *p* < 0.001. Therefore, the association remained statistically significant when uncertainty in the estimation of between-study heterogeneity was incorporated using a *t*-based correction.

Across the 15 leave-one-out models, pooled correlations ranged from *r* = −0.340 to *r* = −0.284. Model-based *I*^2^ ranged from 91.3% to 96.1%. No individual omission changed the inverse direction or statistical significance of the pooled association. The largest reduction in heterogeneity followed the omission of Amiri Amjad et al. [43]. Influence diagnostics identified this study as potentially influential (deleted studentized residual 3.897; DFBETAS 1.059; Cook’s distance 0.562), but its omission strengthened rather than eliminated the pooled inverse association. Full results are presented in Supplementary Tables S7 and S8 and Supplementary Figure S5.

After including three additional studies reporting Spearman coefficients [37,56,58], the combined analysis included 18 studies and 8474 participants and remained significantly inverse (*r* = −0.305; 95% CI −0.395 to −0.211; *p* < 0.001; model-based *I*^2^ = 95.3%). In overlap sensitivity analyses, omission of Zeleníková et al. (2020) [37] yielded *r* = −0.311 (95% CI −0.405 to −0.211; *I*^2^ = 95.5%), and omission of Zeleníková et al. (2021) [58] yielded *r* = −0.309 (95% CI −0.403 to −0.208; *I*^2^ = 95.4%).

When the three Spearman studies were included and Amiri Amjad et al. was excluded, the analysis included 17 studies and 8094 participants, with a pooled *r* = −0.336 (95% CI −0.403 to −0.267; *p* < 0.001; model-based *I*^2^ = 91.1%).

In an additional exploratory analysis, Kendall’s τ from Papastavrou et al. [32] was converted to an equivalent *r* and added to the Pearson and Spearman studies. This model included 19 studies and 8867 participants and yielded *r* = −0.308 (95% CI −0.393 to −0.219; *p* < 0.001; model-based *I*^2^ = 95.0%). Because it combined correlation metrics through an approximate conversion, this analysis was treated as exploratory. The sensitivity-analysis estimates are summarized in Table 4.

Two additional post hoc sensitivity analyses addressed measurement comparability. Restricting the primary dataset to studies using the MISSCARE instrument family yielded *r* = −0.256 (*k* = 9; *N* = 2557; 95% CI −0.409 to −0.089; *I*^2^ = 94.7%). Restricting to PES/NWI-family work-environment measures yielded *r* = −0.289 (*k* = 10; *N* = 4894; 95% CI −0.431 to −0.132; *I*^2^ = 96.8%). When both constructs were simultaneously restricted to the dominant MISSCARE and PES/NWI instrument families, the pooled association was *r* = −0.271 (*k* = 6; *N* = 1788; 95% CI −0.495 to −0.014; *I*^2^ = 96.7%). These restriction analyses preserved the inverse average direction but did not resolve heterogeneity and are exploratory because the joint restriction contains only six studies.

### 3.6. Separate Meta-Analysis of Odds Ratios

Three studies reported adjusted odds ratios that could be displayed in a separate post hoc exploratory synthesis, but they differed materially in exposure categorization, outcome operationalization, analysis level, and adjustment sets [23,35,42]. Accordingly, the pooled OR is retained only as a descriptive robustness summary and is not interpreted as a quantitatively equivalent effect family to the primary correlation model. Study-specific definitions and covariates are detailed in Supplementary Table S14.

The exploratory pooled estimate was OR = 0.381 (95% CI 0.342 to 0.423; *p* < 0.001). Because one large study contributed substantial precision and the three adjusted models were not operationally identical, this narrow confidence interval should not be interpreted as evidence of high certainty or universal comparability. The individual adjusted ORs therefore remain the primary information for interpreting this secondary effect family.

No statistical heterogeneity was detected, *Q*(2) = 0.347; *I*^2^ = 0%. With only three studies and different exposure operationalizations and adjustment sets, this finding should not be interpreted as definitive evidence of homogeneity. The exploratory odds-ratio results are summarized in Table 5.

Park et al. classified practice environments as poor, moderate, or good and reported an adjusted OR of 0.377 (95% CI 0.335 to 0.425) for good versus poor environments for any missed nursing care; the multilevel model controlled for hospital and unit characteristics, including teaching status, hospital size, Magnet status, location, patient case mix, and unit type [35]. Lake et al. reported an adjusted OR of 0.38 (95% CI 0.29 to 0.51) for better versus poor labor-and-delivery work environments for any missed care, controlling for nurse education, RN experience, hospital bed size, technology status, and teaching status while accounting for clustering within hospitals [23]. Zárate-Grajales et al. reported an adjusted OR of 0.43 (95% CI 0.28 to 0.65) for enhanced versus attenuated practice environments using a fractional regression model for an overall missed-care index and a broader set of individual- and workplace-level covariates [42]. These differences support treating the pooled OR as exploratory rather than as a definitive homogeneous summary.

### 3.7. Exploratory Moderator Analyses

Three separate univariable exploratory meta-regression analyses were performed to examine possible sources of heterogeneity.

Year of publication was not a statistically significant moderator, *b* = −0.0004; SE = 0.0175; 95% CI −0.0346 to 0.0339; *p* = 0.984.

The type of missed-care instrument was not a statistically significant moderator (MISSCARE family, *k* = 9; other instruments, *k* = 6; *b* = 0.118; SE = 0.120; 95% CI −0.118 to 0.354; *p* = 0.327). The type of work-environment measure was likewise not significant (PES/NWI family, *k* = 10; other measures, *k* = 5; *b* = 0.041; SE = 0.130; 95% CI −0.213 to 0.295; *p* = 0.753). These binary classifications are deliberately coarse and may mask differences in instrument content, scoring, reliability, and conceptual framework; the analyses therefore do not establish measurement equivalence.

The type of nursing work environment instrument, classified as a PES/NWI-family instrument or another multidimensional measure, was also not statistically significant, *b* = 0.041; SE = 0.130; 95% CI −0.213 to 0.295; *p* = 0.753.

Because only 15 studies were available in the primary model, these analyses were exploratory. Failure to identify a statistically significant moderator does not demonstrate the absence of moderation. The moderator estimates are summarized in Table 6.

Detailed results of the meta-regression analyses are presented in Supplementary Table S2.

### 3.8. Assessment of Potential Publication Bias

The funnel plot of the primary Pearson meta-analysis is presented in Supplementary Figure S4.

Egger’s regression test did not indicate funnel-plot asymmetry (standard-error coefficient = −0.705; SE = 3.880; *t*(13) = −0.182; *p* = 0.859). The Begg-Mazumdar rank-correlation test was also non-significant (Kendall’s τ = 0.105; *p* = 0.627), and the Duval-Tweedie trim-and-fill procedure imputed no missing studies, leaving the pooled estimate unchanged. These findings provide no statistical indication of small-study effects under the applied methods, but they do not demonstrate the absence of publication bias because *k* = 15 and heterogeneity was very high.

The absence of a statistically significant Egger test does not rule out the possibility of selective publication or other causes of asymmetry.

### 3.9. Narrative Synthesis of Additional Evidence

Additional eligible studies were synthesized by effect family because they reported regression, structural-equation, longitudinal, subscale-level, null/mixed, or otherwise non-comparable results rather than an eligible overall Pearson total–total coefficient.

Predominantly inverse findings were reported in adjusted and subscale analyses. Hessels et al. found an inverse standardized coefficient for total PES-NWI (β = −0.666) [33]; Labrague reported significant inverse subscale-level coefficients [28]; Tamayo et al. reported *B* = −0.20 for the overall environment [41]; and Albsoul et al. observed small inverse correlations for two PES-NWI subscales, while no overall total–total coefficient was available [44]. Paiva-Santos et al. and Simonetti et al. likewise reported less missed care in more favorable environments [27,40].

Structural-equation and mediation studies were directionally similar but were not pooled with zero-order correlations. Bruyneel et al. linked work environment with unfinished care through mediation models [46]; Rivaz et al. reported dimension-level SEM effects [47]; and Labrague et al. reported both direct and indirect inverse effects [51]. Zhao et al. contributed an eligible zero-order correlation to the primary model, while its mediation analysis provided additional explanatory evidence [52].

Longitudinal evidence was directionally concordant. Lake et al. reported that improved work environments were associated with less missed care over time [24], Liu et al. linked changes in the nursing work environment and non-professional tasks with care left undone [38], and Lu et al. linked the T1 work environment with T3 missed nursing care (*r* = −0.502) [53].

Null, mixed, and non-extractable overall findings were also retained. Bartoníčková et al. reported no significant overall association [39]. Viana Santana et al., Giannarou et al., Pragosa et al., and Caillet et al. did not report an eligible overall total–total estimate [50,55,57,59]. The Giannarou sample also combined registered nurses and nursing assistants without a separate eligible total effect for registered nurses [55].

Several reports were informative only at the level of activities, subscales, or non-comparable regression parameters. Özmen and Arslan Yürimezoğlu reported logistic models for individual missed-care activities [49], while Caponnetto et al. reported quantile-regression effects for selected practice-environment subscales in home care [60]. These findings were retained narratively without being treated as direct replications of the primary total–total association.

Taken together, the non-pooled evidence showed a predominantly inverse pattern but also included null, mixed, and non-extractable overall results. Supplementary Table S3 presents every included study, its reported effect, and the synthesis category used.

## 4. Discussion

### 4.1. Main Findings

The primary meta-analysis showed an average inverse association between the nursing work environment and missed nursing care. Across 15 studies and 7100 participants, the pooled coefficient was *r* = −0.301. However, this mean estimate must be interpreted together with *I*^2^ = 95.7% and the prediction interval of −0.641 to 0.139. The confidence interval around the pooled mean quantifies uncertainty in the average association, whereas the prediction interval reflects expected between-setting variation in a new comparable study. Because the latter crosses zero, the evidence supports an inverse association on average but not a universal negative effect across all clinical and health-system contexts.

The pooled magnitude was small to moderate. Its practical importance should be interpreted together with the complex, multilevel determinants of missed care and the prediction interval crossing the null; the estimate is an average across heterogeneous settings rather than a universal effect.

The primary pattern was predominantly inverse: 14 of 15 studies reported the expected direction, while Amiri Amjad et al. reported the only positive coefficient [43].

The additional robustness analyses supported this conclusion. The Hartung–Knapp correction retained a statistically significant association, and sequential omission of every study produced consistently negative and statistically significant pooled estimates. Thus, the overall finding was not dependent on any single study, although the magnitude of the association and heterogeneity changed most after omission of Amiri Amjad et al.

The separate odds-ratio meta-analysis was directionally concordant with the primary analysis but remained exploratory. The pooled OR suggested lower odds of missed care in more favorable environments, but only three studies contributed, and they differed in exposure definitions and covariate adjustment [23,35,42]. This pattern is compatible with earlier reviews identifying workload, staffing, resources, leadership, autonomy, communication, and organizational support as antecedents of incomplete care [9,10,11].

### 4.2. Comparison with Previous Evidence

The findings are consistent with previous reviews describing relationships between organizational nursing environments and missed or unfinished care [9,10,11]. The present synthesis extends that evidence by quantifying a strict total-score Pearson association, separating alternative effect-size families, and explicitly examining prediction intervals, influential observations, risk-of-bias restrictions, and instrument-family restrictions. These analyses strengthen transparency about the average association but do not convert observational associations into evidence of causation.

The pooled *r* = −0.301 quantifies the average overall association across countries and health systems. The high heterogeneity and prediction interval show that it should not be interpreted as a setting-invariant effect.

The results are theoretically consistent with the conceptual framework of the PES-NWI, which encompasses nurse participation in hospital affairs, nursing foundations of quality care, nurse manager ability, leadership and support, staffing and resource adequacy, and nurse-physician collegiality [6,7]. Each of these dimensions can influence whether nurses have sufficient time, authority, resources, professional support, and opportunities for collaboration to provide comprehensive care.

Subscale findings were also predominantly inverse. Nursing foundations for quality of care was the only significant independent predictor in Babaei et al. [29] and showed the strongest significant PES-NWI subscale correlation in Albsoul et al. [44]. In pediatric units, staffing/resource adequacy, quality foundations, manager support, and collegial relationships predicted selected missed activities [49].

Taken together, these findings suggest that missed care is not simply a consequence of a shortage of nurses. It reflects a broader organizational system that includes professional standards, leadership, participation, teamwork, communication, decision-making, and the institution’s ability to support the delivery of comprehensive care.

### 4.3. High Heterogeneity Across Studies

The extremely high heterogeneity is clinically and methodologically important rather than a secondary statistical detail. Plausible sources include differences in countries and health systems, hospital and unit settings, cross-sectional versus longitudinal designs, nurse and patient populations, staffing and workload context, outcome definitions, recall periods, instrument content and scoring, analytical adjustment, and risk-of-bias domains such as confounding. The post hoc instrument-family restrictions and risk-of-bias sensitivity analyses did not eliminate heterogeneity, indicating that no single measured feature adequately explains the observed between-study variation.

Several factors could contribute to the heterogeneity.

First, different instruments were used to assess the nursing work environment. Although a large number of studies used the PES-NWI or other instruments from the NWI family, others used separate work environment scales that may not capture exactly the same organizational dimensions or use the same scoring methods.

Second, missed care has been operationalized through different conceptual and measurement traditions, including MISSCARE, care left undone, unfinished nursing care, implicit rationing, and other measures. Differences in recall period, number of activities assessed, response scales, scoring direction, and method of calculating the total score may contribute to variations in correlations [1,2,3].

Third, clinical settings varied considerably. These included general medical and surgical units, neonatal and pediatric care, intensive care units, emergency departments, labor and delivery wards, and other specialized settings. The likelihood and type of missed care may vary depending on care demands, patient severity, staffing patterns, workflow, and professional responsibilities.

Fourth, the studies were conducted in different countries and healthcare systems. Differences in nurse-patient ratios, professional autonomy, scope of work, hierarchical structure, health financing, resource availability, cultural norms, and willingness to report omissions may influence both the frequency of missed care and its association with organizational conditions.

Fifth, both main constructs were most often self-reported by the same participants. Such an approach may increase the risk of shared methodological variance and responses influenced by cultural patterns. Nurses working in a supportive safety culture may be more willing to admit to failures, while employees in punitive environments may underreport them.

Exploratory meta-regression analyses did not show a statistically significant moderating role for year of publication, type of missed care assessment instrument, or type of work environment assessment instrument. However, these results do not prove that moderation does not exist. With only 15 primary studies, the statistical power of the meta-regression is limited, and the broad binary categorization of instruments may obscure more subtle methodological differences.

Therefore, high heterogeneity should not be considered a reason to reject the pooled estimate, but it does require caution in interpretation. The pooled *r* represents the average association across different contexts, not the universal effect size that can be expected in any health care system.

The leave-one-out findings further indicate that the heterogeneity was not generated by only one extreme study. Even after the most influential study was omitted, model-based *I*^2^ remained 91.3%. The observed heterogeneity therefore appears to reflect genuine variation across several clinical, organizational, national, and measurement contexts rather than a single anomalous result.

### 4.4. Study with a Directionally Different Result

The study by Amiri Amjad et al. was the only primary study with a positive coefficient (*r* = 0.285) and was identified as influential by the diagnostic analyses. The coefficient was retained exactly as published because there was no verified scoring or reporting error that would justify reversing it. Its different direction may reflect study-specific measurement, sample, cultural, or organizational context, but the available data do not establish the mechanism. Excluding this study post hoc strengthened the pooled inverse association to *r* = −0.340 and reduced *I*^2^ from 95.7% to 91.3%; importantly, the main conclusion regarding the average direction was unchanged.

### 4.5. Implications for Nursing Practice and Management

The results have practical implications for nursing management, but they identify plausible organizational targets rather than proven causal interventions. Staffing and resource adequacy, nurse-manager leadership and support, professional participation, autonomy, interdisciplinary collaboration, workload management, and psychological safety are components of the organizational context that were repeatedly associated with missed-care outcomes across the evidence base. The present review does not demonstrate that changing any one of these factors will necessarily reduce missed care.

These factors may therefore be considered priorities for organizational assessment and hypothesis-driven intervention studies. Decisions to modify staffing models, leadership processes, professional participation, resources, training, or workload should be evaluated prospectively, ideally with longitudinal, quasi-experimental, or interventional designs capable of testing whether changes in the work environment precede and cause changes in missed nursing care.

The results of this meta-analysis therefore empirically support the assumption that organizational characteristics at the structure level are measurably related to health care processes.

Because the evidence was predominantly observational and cross-sectional, causality cannot be inferred. The findings are more accurately described as an average inverse association that is compatible with Donabedian’s framework rather than proof that organizational structure causes changes in missed care.

From a management perspective, interventions should not be limited to increasing the number of nurses, although adequacy of staffing and resources remains central. Improvements may also require stronger nursing leadership, greater participation of nurses in organizational decision-making, clear professional standards, better interdisciplinary communication, greater psychological safety, sufficient material resources, continuing professional education, and effective workload management.

Regular, simultaneous measurement of the nursing work environment and missed care could also serve as an organizational early warning system. Deteriorating work environment scores with concomitant increases in missed care could help identify units where quality and patient safety are compromised before more serious adverse outcomes occur.

### 4.6. Strengths of the Study

This review has several important strengths.

The primary meta-analysis included 15 studies and 7100 participants, representing a stronger quantitative basis than smaller initial models.

The main analysis was deliberately limited to Pearson correlations between the total measures of both constructs, thereby increasing the conceptual and statistical comparability of the primary effect size.

Sensitivity analyses included exclusion of the directionally different study, Hartung–Knapp adjustment, sequential leave-one-out analysis, formal influence diagnostics, Spearman correlations, overlap checks for the two Zeleníková reports, and an exploratory converted Kendall coefficient. The average inverse conclusion was robust to these analyses, although heterogeneity remained very high.

Different families of effect sizes were not inappropriately combined. Odds ratios were analyzed separately from correlations, while regression coefficients, structural equation modeling effects, longitudinal effects, and individual subscale scores were synthesized narratively.

The systematic review included 40 unique studies from different countries, health systems, and clinical settings, including very recent studies published in 2026.

In addition, post hoc robustness analyses included Hartung-Knapp adjustment, leave-one-out and influence diagnostics, risk-of-bias restrictions, instrument-family restrictions, overlap sensitivity, exploratory meta-regression, and several small-study-effect assessments. Their post hoc status is explicitly identified so that they are interpreted as robustness and hypothesis-generating analyses rather than prespecified confirmatory tests.

### 4.7. Limitations

Several limitations need to be considered.

First, heterogeneity in the primary analysis was very high. Fourteen of 15 primary studies reported an inverse direction, but magnitudes varied considerably, and the prediction interval crossed the null. The pooled coefficient is therefore an average across contexts rather than a universal effect.

Second, most of the primary studies were cross-sectional. Therefore, neither temporal sequence nor causality can be established. It is theoretically plausible that an adverse work environment contributes to missed care, but it is also possible that high levels of missed care worsen perceptions of the work environment.

Third, both exposure and outcome were most often measured by self-report questionnaires completed by the same participants. This raises the possibility of shared methodological bias, socially desirable responses, and under- or over-reporting of care lapses.

Fourth, different instruments and scoring methods were used. Although the primary analysis was limited to total–total Pearson correlations and the directions of the effects were harmonized, differences in the operationalization of both constructs remained.

Fifth, the Amiri Amjad et al. study was identified as potentially influential and affected the pooled magnitude and heterogeneity. Nevertheless, systematic leave-one-out analysis showed that no individual study changed the direction or statistical significance of the result, and heterogeneity remained very high after every omission.

Sixth, not all eligible studies could be pooled. Albsoul et al. reported only PES-NWI subscale-to-total correlations [44], while Giannarou et al. did not report an overall total–total correlation and included a mixed registered-nurse/nursing-assistant sample [55].

Seventh, the secondary odds-ratio synthesis included only three studies whose exposure categories, outcome definitions, analysis levels, and covariate sets differed. Its pooled estimate is therefore presented only as exploratory; the individual adjusted ORs are more directly interpretable than the pooled value.

Eighth, the post hoc meta-regression analyses were based on only 15 studies and had limited statistical power. The categorical moderators were also coarse (MISSCARE family versus other and PES/NWI family versus other), with heterogeneous instruments grouped in the “other” categories. Non-significant moderator tests therefore do not demonstrate that the measurement approach or publication year are unrelated to heterogeneity.

Ninth, funnel-plot, Egger, rank-correlation, and trim-and-fill analyses were conducted with only 15 studies in the presence of very high heterogeneity. Their negative findings do not exclude publication bias or other small-study effects.

Tenth, there was no prospective publicly available protocol. The core eligibility criteria, total-score Pearson primary analysis, random-effects REML model, and direction harmonization were specified before inspection of the pooled results, but the additional robustness and exploratory analyses were introduced post hoc. The OSF record was created retrospectively on 25 August 2026, after screening, extraction, and primary analysis. This sequence limits the protection against data-informed analytical choices and is reported explicitly.

Eleventh, one potentially relevant report could not be retrieved despite institutional, publisher, Google Scholar, repository, and reference-list searches [20]. Availability bias, therefore, cannot be excluded.

Twelfth, CINAHL was not searched because institutional access was unavailable. PubMed, Scopus, Web of Science, APA PsycInfo, citation tracking, reference-list searching, Google Scholar, and targeted searches provided broad coverage, but these sources cannot be assumed to fully compensate for a nursing-specific database. Relevant nursing studies may therefore have been missed.

Thirteenth, a formal GRADE or other certainty-of-evidence rating was not conducted. Such assessment is not straightforward for this heterogeneous synthesis of predominantly observational studies and multiple effect-size families. Consequently, statistical precision of the pooled mean should not be equated with high certainty of evidence. Confidence in broad applicability is limited by very high heterogeneity, the prediction interval crossing zero, measurement diversity, residual confounding, and the predominance of cross-sectional designs.

Fourteenth, the sampling variance SE = √[1/(*N* − 3)] treats observations as independent. Nurses were often clustered within units or hospitals, but intraclass correlations and design effects were generally unavailable. Consequently, study-level precision may be overstated where published correlations did not account for clustering.

Fifteenth, Zeleníková et al. [37,58] arose from a related research context, and partial participant overlap could not be excluded from the available reports. Omitting either report from the Pearson-plus-Spearman model left the pooled estimate essentially unchanged (*r* = −0.311 and *r* = −0.309, respectively), but both should not be interpreted as fully independent confirmatory replications.

### 4.8. Future Research

Future research should more frequently employ longitudinal, quasi-experimental, and interventional designs to establish temporal ordering and test whether modifying specific work-environment components causes changes in missed nursing care. Such designs are necessary before organizational factors identified in this review can be considered evidence-based causal interventions.

Future reports should present total scores, subscale scores, and complete zero-order correlation matrices when both constructs are measured. Missing overall total–total coefficients unnecessarily limit quantitative synthesis.

Greater standardization of outcome measures would further improve comparability. Although the use of different validated instruments reflects different conceptual traditions, differences in scoring direction, item structure, recall period, and response scales contribute to heterogeneity.

Future studies should examine whether the association differs by clinical setting, patient-to-nurse ratio, patient severity, country-level economic development, professional autonomy, leadership model, and organizational safety culture. These moderators are theoretically plausible but could not be reliably tested in this meta-analysis due to the limited number of comparable studies.

Research is also needed that will use independent or more objective indicators of missed care, triangulation with medical documentation or observation, and multilevel models that separate effects at the level of the nurse, unit, hospital, and health system.

## 5. Conclusions

On average, a more supportive nursing work environment was associated with less missed nursing care.

Across 15 studies and 7100 participants, the pooled Pearson correlation was *r* = −0.301. After exclusion of the only directionally different study, the magnitude of the association strengthened to *r* = −0.340.

The average inverse result persisted under Hartung–Knapp and all 15 leave-one-out models and was directionally concordant with the separate exploratory odds-ratio synthesis. These analyses support the robustness of the average finding but do not remove the uncertainty represented by very high heterogeneity.

However, the extremely high heterogeneity indicates that the magnitude of the association varies considerably across health systems, clinical settings, measurement instruments, and study designs. The pooled result should therefore be interpreted as an average association and not as a universal effect.

Staffing and resource adequacy, nursing leadership, professional participation, autonomy, interdisciplinary collaboration, workload management, and organizational support are plausible targets for assessment and future intervention research because they form part of the organizational context represented in the included evidence. However, the present synthesis is predominantly observational and supports association rather than causal efficacy; whether modifying these factors reduces missed nursing care requires prospective interventional evidence.

## Figures and Tables

**Table 1:** Detailed eligibility criteria and summary of the study screening and selection process.

Criterion/Screening Stage	Definition/*n*
Eligibility criteria for inclusion in the systematic review	
Review population	Direct-care nurses; mixed-workforce reports considered narratively only when relevant to the review question.
Exposure	Multidimensional nursing work, practice, or professional environment.
Outcome	Missed, incomplete, unfinished, omitted, implicitly rationed, or otherwise unperformed nursing care.
Eligible designs	Quantitative cross-sectional, longitudinal, or other observational studies reporting an association between exposure and outcome.
Eligibility criteria for inclusion in the primary meta-analysis	
Primary meta-analysis eligibility	Total-score Pearson correlation between overall nursing work/practice environment and overall missed nursing care.
Studies included in the systematic review but not eligible for the primary meta-analysis	Non-Pearson metrics; subscale-only/individual-activity effects; regression/SEM/longitudinal effects without an eligible total-score Pearson *r*; mixed-workforce effects without separable nurse results. Such studies remained included in the systematic review when the systematic-review eligibility criteria were otherwise met, but did not enter the primary Pearson meta-analysis.
Study screening and selection process	
Database records identified	292
Duplicates removed	205
Unique records screened	87
Records excluded at title/abstract stage	47
Database reports sought	40
Database reports not retrieved	1
Full-text reports assessed from database searching	39
Additional reports assessed	7
Full-text reports assessed across all routes	46
Full-text reports excluded from the systematic review	6
Studies included in the systematic review	40
Studies included in the systematic review but not included in the primary Pearson meta-analysis	25
Studies included in the primary Pearson meta-analysis	15

**Table 2:** Studies included in the primary Pearson correlation meta-analysis.

Study	Country	*N*	Practice-Environment Measure	Missed-Care Measure	Pearson’s *r*
Menard, 2014 [31]	USA	557	PES-NWI/practice-environment measure	MISSCARE Survey	−0.510 †
Kim et al., 2018 [34]	Republic of Korea	186	PES-NWI	MISSCARE Survey	−0.430
Smith et al., 2018 [25]	USA	233	PES-NWI composite	MISSCARE Survey	−0.200
Ibrahim and Abohabieb, 2020 [36]	Egypt	136	Nursing work environment measure	Missed nursing care measure	−0.200
Smith et al., 2020 [26]	Australia	383	NWI-R:A	Care left undone	−0.360
Campbell et al., 2020 [21]	USA	950	PES-NWI	PIRNCA	−0.423
Liu et al., 2018 [22]	China	1542	PES-NWI	Nursing care left undone	−0.080
Elpasiony and Abd-Elmoghith, 2023 [45]	Egypt	200	Nursing work environment measure	MISSCARE Survey	−0.229
Amiri Amjad et al., 2023 [43]	Iran	380	PES-NWI	MISSCARE Survey	0.285
Babaei et al., 2024 [29]	Iran	255	NWI-PES	MISSCARE Survey	−0.180
El-Gazar et al., 2024 [30]	Egypt	231	PES-NWI	PIRNCA	−0.380
Çiriş Yildiz et al., 2024 [48]	Türkiye	433	Nursing work environment measure	Missed nursing care measure	−0.249
Zhao et al., 2026 [52]	China	535	Nursing Work Environment Scale	Missed Nursing Care Scale	−0.399
Lu et al., 2026 [53]	China	902	Clinical Nursing Work Environment Scale	Missed nursing care measure	−0.502
Kim and Uhm, 2026 [54]	Republic of Korea	177	PES-NWI-5	MISSCARE Survey	−0.510

Note: *N* = sample size; *r* = Pearson correlation coefficient; PES-NWI = Practice Environment Scale of the Nursing Work Index; NWI-R:A = Nursing Work Index–Revised: Australian version. † The sign of the Menard coefficient was reversed for pooling because the study-specific scoring orientation was opposite to the review convention; the magnitude was unchanged. The positive Amiri Amjad et al. coefficient was retained as published. Full study-level scoring and harmonization decisions are reported in Supplementary Table S10.

**Table 3:** Primary meta-analysis of the association between the nursing practice environment and missed nursing care.

*k*	Total *N*	Pooled *r*	95% CI	*p*	*Q*	*I* ^2^	τ^2^	95% Prediction Interval
15	7100	−0.301	−0.402 to −0.192	<0.001	361.599	95.7%	0.049	−0.641 to 0.139

Note: *k* = number of studies; *N* = total sample size; *r* = pooled Pearson correlation coefficient; CI = confidence interval; *p* = statistical significance; *Q* = Cochran’s heterogeneity statistic; *I*^2^ = model-based percentage of total variability attributable to between-study heterogeneity, as returned by metafor; τ^2^ = estimated between-study variance.

**Table 4:** Sensitivity analyses of the association between the nursing practice environment and missed nursing care.

Analysis	*k*	Total *N*	Pooled *r*	95% CI	*p*	*I* ^2^
Primary Pearson model	15	7100	−0.301	−0.402 to −0.192	<0.001	95.7%
Pearson model excluding Amiri Amjad et al.	14	6720	−0.340	−0.413 to −0.263	<0.001	91.3%
Pearson + Spearman	18	8474	−0.305	−0.395 to −0.211	<0.001	95.3%
Pearson + Spearman, excluding Amiri Amjad	17	8094	−0.336	−0.403 to −0.267	<0.001	91.1%
Pearson + Spearman + converted Kendall τ	19	8867	−0.308	−0.393 to −0.219	<0.001	95.0%
Risk of bias: no explicit N in mapped key domains	14	6717	−0.296	−0.405 to −0.179	<0.001	96.0%
Risk of bias: all mapped key domains rated Y	11	6001	−0.358	−0.443 to −0.266	<0.001	93.2%
MISSCARE-family restriction	9	2557	−0.256	−0.409 to −0.089	0.003	94.7%
PES/NWI-family restriction	10	4894	−0.289	−0.431 to −0.132	<0.001	96.8%
MISSCARE + PES/NWI joint restriction	6	1788	−0.271	−0.495 to −0.014	0.039	96.7%

Note: *k* = number of studies; *N* = total sample size; *r* = pooled correlation coefficient; CI = confidence interval; *p* = statistical significance; *I*^2^ = model-based between-study heterogeneity returned by metafor. The model, including converted Kendall τ, is exploratory because it relies on an approximate conversion between different correlation metrics.

**Table 5:** Exploratory meta-analysis of odds ratios for missed nursing care according to the nursing practice environment.

*k*	Pooled OR	95% CI	*p*	*Q*	*I* ^2^
3	0.381	0.342 to 0.423	<0.001	0.347	0%

Note: *k* = number of studies; OR = odds ratio; CI = confidence interval; *p* = statistical significance; *Q* = Cochran’s heterogeneity statistic; *I*^2^ = between-study heterogeneity.

**Table 6:** Exploratory meta-regression analyses.

Moderator	*b*	SE	95% CI	*p*	Proportion of Between-Study Heterogeneity Explained
Year of publication	−0.0004	0.0175	−0.0346 to 0.0339	0.984	0%
Missed-care instrument type (MISSCARE *k* = 9; other *k* = 6)	0.118	0.120	−0.118 to 0.354	0.327	0%
Practice-environment instrument type (PES/NWI *k* = 10; other *k* = 5)	0.041	0.130	−0.213 to 0.295	0.753	0%

Note: *b* = meta-regression coefficient on the Fisher *z* scale; SE = standard error; CI = confidence interval; *p* = statistical significance. Each moderator was examined in a separate univariable exploratory model because only 15 studies were available in the primary Pearson model.

## Data Availability

Analysis-ready study-level data are provided in Supplementary Dataset S1 and in the accompanying Excel S1 workbook. The complete R script and R session information are provided as Supplementary Files S1 and S2.

## Supplementary Materials

The following supporting information can be downloaded at: https://www.mdpi.com/article/10.3390/nursrep16090336/s1, Table S1, Complete database-specific search strategies [13]; Table S2, Exploratory meta-regression analyses; Table S3, Characteristics, extracted findings, synthesis category, and reason for non-inclusion in the primary Pearson meta-analysis [21,22,23,24,25,26,27,28,29,30,31,32,33,34,35,36,37,38,39,40,41,42,43,44,45,46,47,48,49,50,51,52,53,54,55,56,57,58,59,60]; Tables S4A and S4B, Detailed JBI appraisal matrices for analytical cross-sectional studies and longitudinal/cohort studies, respectively [61,62]; Table S5, Full-text reports excluded, with reasons [14,15,16,17,18,19]; Table S6, Reports sought but not retrieved [20]; Table S7, Leave-one-out analysis of the primary Pearson meta-analysis; Table S8, Influence diagnostics for the primary Pearson meta-analysis; Table S9, Sensitivity analysis for possible overlap between the Zeleníková 2020 and 2021 reports; Table S10, Study-level scoring direction and effect harmonization in the primary Pearson meta-analysis; Table S11, Post-hoc risk-of-bias sensitivity analyses; Table S12, Post-hoc instrument-family sensitivity analyses; Table S13, Post-hoc assessment of funnel asymmetry and small-study effects; Table S14, Comparability of studies in the post-hoc exploratory adjusted-odds-ratio synthesis; Figure S1, Forest plot of the primary Pearson correlation meta-analysis; Figure S2, Forest plot of the sensitivity analysis excluding Amiri Amjad et al.; Figure S3, Forest plot of the separate odds-ratio meta-analysis; Figure S4, Funnel plot for the primary Pearson correlation meta-analysis; Figure S5, Influence diagnostics for the primary Pearson meta-analysis; Figure S6, Baujat plot for the primary Pearson meta-analysis; Figure S7, Trim-and-fill funnel plot for the primary Pearson meta-analysis; Figure S8, Forest plot of the strict risk-of-bias sensitivity analysis retaining only studies rated Y in all mapped key JBI domains; Figure S9, Forest plot of the joint MISSCARE-family and PES/NWI-family instrument restriction; Dataset S1, Study-level meta-analysis dataset; File S1, complete R analysis script, and File S2, R session information [63,64]; and the analysis-ready Excel workbook and exported numerical results; File S3, PRISMA 2020 checklist; File S4, Complete R analysis qprofile; Excel S1, Nursing environment missed care R studio.

## Public Involvement Statement

No members of the public were involved in the design, conduct, reporting, or dissemination plans of this research.

## Guidelines and Standards Statement

This systematic review and meta-analysis was reported in accordance with PRISMA 2020 [12], with search reporting additionally informed by PRISMA-S [13].
