# Supplementary material for: The Association Between the Nursing Work Environment and Missed Nursing Care: A Systematic Review and Meta-Analysis
Source: Nurs Rep. 2026 Sep 15;16(9):336. doi: 10.3390/nursrep16090336 (PMC13610344; doi:10.3390/nursrep16090336)
Supplement: Supplementary file 1 [file nursrep-16-00336-s001.zip › File S1 R CODE.pdf]

File S1. Complete R analysis script — reviewer revision with new lines highlighted

Yellow highlighting marks lines added or revised relative to the supplied V5 reviewer-analysis script. The executable .R file is provided separately.

```
# COMPLETE REPRODUCIBLE META-ANALYSIS
# REVIEWER REVISION: ANALYTIC DECISION STATUS AND TIMELINE
# Prespecified before inspection of pooled results:
#   (1) eligibility criteria;
#   (2) primary total-score Pearson correlation meta-analysis;
#   (3) random-effects model with REML estimation; and
#   (4) effect-direction harmonization.
# Added post hoc as robustness/exploratory analyses:
#   Hartung-Knapp, leave-one-out, Amiri exclusion, Pearson+Spearman,
#   converted Kendall tau, overlap sensitivity, risk-of-bias sensitivity,
#   instrument-family sensitivity, meta-regression, influence diagnostics,
#   Egger/rank-correlation tests, and trim-and-fill.
# Review timeline:
#   Initial search/screening: 15 March-15 June 2026
#   Data extraction: 20 May-15 July 2026
#   Final search update: 13 July 2026
#   Statistical analysis: 16-22 July 2026
#   Retrospective OSF registration: 25 August 2026; DOI 10.17605/OSF.IO/J62D3
# Screening reviewers: M.M. and I.M.
# Data extraction: M.M.; verification of all extracted variables: I.M.
# JBI risk-of-bias appraisal: M.M. and N.F.
# Deduplication: Zotero first, followed by DOI/PMID/title and manual checks
# in the screening workbook.

# Nursing work environment and missed nursing care
# Input: NURSING_ENVIRONMENT_MISSED_CARE_R_STUDIO_SUBMISSION_FINAL.xlsx
#
# Run the entire script in a fresh RStudio session. After the Excel file is
# selected manually, the script creates R_RESULTS_V5_REVIEWER_ANALYSES in the
# same folder and saves all numeric results, figures, and session details there.

message("RUNNING COMPLETE SCRIPT V5: reviewer-requested sensitivity analyses included.")

required_packages <- c("readxl", "metafor", "openxlsx")
missing_packages <- required_packages[
  !vapply(required_packages, requireNamespace, logical(1), quietly = TRUE)
]
if (length(missing_packages) > 0) {
  install.packages(
    missing_packages,
    repos = "https://cloud.r-project.org",
    dependencies = TRUE
  )
}

suppressPackageStartupMessages({
  library(readxl)
  library(metafor)
  library(openxlsx)
})

```

```
options(stringsAsFactors = FALSE, digits = 7)

# ---------------------------------------------------------------------------
# 1. FILES AND OUTPUT FOLDER
# ---------------------------------------------------------------------------

message("Select NURSING_ENVIRONMENT_MISSED_CARE_R_STUDIO_SUBMISSION_FINAL_REVISED.xlsx")
excel_file <- file.choose()
input_directory <- dirname(normalizePath(excel_file))
output_directory <- file.path(
  input_directory,
  "R_RESULTS_V5_REVIEWER_ANALYSES"
)
dir.create(output_directory, showWarnings = FALSE, recursive = TRUE)

# ---------------------------------------------------------------------------
# 2. IMPORT AND VALIDATION
# ---------------------------------------------------------------------------

primary <- read_excel(excel_file, sheet = "PRIMARY_15", skip = 3)
all_correlations <- read_excel(excel_file, sheet = "ALL_CORRELATIONS", skip = 3)
odds_ratios <- read_excel(excel_file, sheet = "OR_3", skip = 3)

primary <- primary[!is.na(primary$Study) & primary$Primary_include %in% TRUE, ]
all_correlations <- all_correlations[!is.na(all_correlations$Study), ]
odds_ratios <- odds_ratios[!is.na(odds_ratios$Study), ]

# Convert logical columns reliably if Excel imports them as numbers.
logical_columns <- c(
  "Primary_Pearson", "Pearson_plus_Spearman",
  "Pearson_Spearman_no_Amiri", "All_converted"
)
for (column_name in logical_columns) {
  all_correlations[[column_name]] <- as.logical(all_correlations[[column_name]])
}

stopifnot(nrow(primary) == 15L)
stopifnot(sum(primary$N) == 7100L)
stopifnot(primary$N[grepl("Amiri Amjad", primary$Study)] == 380L)
stopifnot(all(abs(primary$r_harmonized) < 1))
stopifnot(all(primary$N > 3))
stopifnot(sum(all_correlations$Original_metric == "Spearman rho") == 3L)
stopifnot(any(grepl("Zeleníková et al., 2021", all_correlations$Study)))
stopifnot(nrow(odds_ratios) == 3L)

primary$yi <- atanh(primary$r_harmonized)
primary$vi <- 1 / (primary$N - 3)
primary$sei <- sqrt(primary$vi)
primary$Year_centered_2023 <- primary$Year - 2023

# Helper for consistent back-transformed output.
model_summary_r <- function(analysis_name, model, total_n, use_t = FALSE) {
  prediction <- predict(model, transf = transf.ztor)
  data.frame(
    Analysis = analysis_name,
    k = model$k,
    Total_N = total_n,
```

```
    Pooled_r = as.numeric(transf.ztor(model$b)),
    CI_lower = as.numeric(transf.ztor(model$ci.lb)),
    CI_upper = as.numeric(transf.ztor(model$ci.ub)),
    Test_statistic = as.numeric(model$zval),
    df = if (use_t) model$k - model$p else NA_integer_,
    p_value = as.numeric(model$pval),
    Q = as.numeric(model$QE),
    Q_df = model$k - model$p,
    Q_p = as.numeric(model$QEp),
    I2_percent = as.numeric(model$I2),
    tau2 = as.numeric(model$tau2),
    PI_lower = as.numeric(prediction$pi.lb),
    PI_upper = as.numeric(prediction$pi.ub),
    stringsAsFactors = FALSE
  )
}

# Fit a REML model to an explicitly defined subset of the 15 primary studies.
# Returning the model and the selected data makes all study-count checks
# transparent and prevents accidental reuse of the wrong subset.
fit_primary_subset <- function(selection, analysis_name) {
  if (!is.logical(selection) || length(selection) != nrow(primary)) {
    stop("Primary-study subset must be a logical vector of length 15.")
  }
  if (anyNA(selection)) {
    stop("Primary-study subset contains missing inclusion indicators.")
  }
  selected <- primary[selection, ]
  if (nrow(selected) < 2L) {
    stop("At least two studies are required for a subset meta-analysis.")
  }
  fitted_model <- rma(
    yi = yi,
    vi = vi,
    data = selected,
    method = "REML",
    test = "z"
  )
  list(
    model = fitted_model,
    data = selected,
    summary = model_summary_r(
      analysis_name,
      fitted_model,
      sum(selected$N)
    )
  )
}

# ---------------------------------------------------------------------------
# 3. PRIMARY RANDOM-EFFECTS MODEL (REML)
# ---------------------------------------------------------------------------

model_primary <- rma(
  yi = yi,
  vi = vi,
  data = primary,
```

```
  method = "REML",
  test = "z"
)

primary_summary <- model_summary_r(
  "Primary Pearson REML",
  model_primary,
  sum(primary$N)
)

# Q-profile confidence interval for tau2 and derived model-based I2 interval.
tau2_qprofile_CI <- confint(model_primary, type = "QP")
tau2_qprofile_table <- as.data.frame(tau2_qprofile_CI$random)

# ---------------------------------------------------------------------------
# 4. HARTUNG-KNAPP SENSITIVITY MODEL
# ---------------------------------------------------------------------------

model_HK <- rma(
  yi = yi,
  vi = vi,
  data = primary,
  method = "REML",
  test = "knha"
)

Hartung_Knapp_summary <- model_summary_r(
  "REML with Hartung-Knapp adjustment",
  model_HK,
  sum(primary$N),
  use_t = TRUE
)

# ---------------------------------------------------------------------------
# 5. CORRELATION-METRIC SENSITIVITY ANALYSES
# ---------------------------------------------------------------------------

all_correlations$analysis_r <- all_correlations$Analysis_r
is_Kendall <- all_correlations$Original_metric == "Kendall tau"
all_correlations$analysis_r[is_Kendall] <- sin(
  pi * all_correlations$Raw_effect[is_Kendall] / 2
)
all_correlations$yi <- atanh(all_correlations$analysis_r)
all_correlations$vi <- 1 / (all_correlations$N - 3)

fit_correlation_subset <- function(flag_column, analysis_name) {
  selected <- all_correlations[which(all_correlations[[flag_column]]), ]
  fitted_model <- rma(
    yi = yi,
    vi = vi,
    data = selected,
    method = "REML",
    test = "z"
  )
  list(
    model = fitted_model,
    data = selected,
```

```r
    summary = model_summary_r(
      analysis_name,
      fitted_model,
      sum(selected$N)
    )
  )
}

primary_check <- fit_correlation_subset(
  "Primary_Pearson",
  "Primary Pearson check"
)

data_without_Amiri <- primary[!grepl("Amiri Amjad", primary$Study), ]
model_without_Amiri <- rma(
  yi = yi,
  vi = vi,
  data = data_without_Amiri,
  method = "REML",
  test = "z"
)
without_Amiri_summary <- model_summary_r(
  "Pearson excluding Amiri Amjad",
  model_without_Amiri,
  sum(data_without_Amiri$N)
)

Pearson_plus_Spearman <- fit_correlation_subset(
  "Pearson_plus_Spearman",
  "Pearson plus three Spearman"
)

Pearson_Spearman_without_Amiri <- fit_correlation_subset(
  "Pearson_Spearman_no_Amiri",
  "Pearson plus three Spearman excluding Amiri Amjad"
)

all_converted_correlations <- fit_correlation_subset(
  "All_converted",
  "Pearson plus three Spearman plus converted Kendall tau"
)

sensitivity_summary <- rbind(
  without_Amiri_summary,
  Pearson_plus_Spearman$summary,
  Pearson_Spearman_without_Amiri$summary,
  all_converted_correlations$summary
)


# Sensitivity analyses for possible partial sample overlap between the two
# Zelenikova reports. Each model retains the 15 Pearson studies plus the other
# two Spearman studies.
fit_overlap_sensitivity <- function(study_pattern, analysis_name) {
  selected <- Pearson_plus_Spearman$data[
    !grepl(study_pattern, Pearson_plus_Spearman$data$Study),
  ]
```

```
  fitted_model <- rma(
    yi = yi,
    vi = vi,
    data = selected,
    method = "REML",
    test = "z"
  )
  model_summary_r(analysis_name, fitted_model, sum(selected$N))
}

overlap_sensitivity_summary <- rbind(
  fit_overlap_sensitivity(
    "Zeleníková et al., 2020",
    "Pearson plus Spearman excluding Zelenikova 2020"
  ),
  fit_overlap_sensitivity(
    "Zeleníková et al., 2021",
    "Pearson plus Spearman excluding Zelenikova 2021"
  )
)

# Hard checks against the locked study counts and participant totals.
stopifnot(Pearson_plus_Spearman$model$k == 18L)
stopifnot(sum(Pearson_plus_Spearman$data$N) == 8474L)
stopifnot(Pearson_Spearman_without_Amiri$model$k == 17L)
stopifnot(sum(Pearson_Spearman_without_Amiri$data$N) == 8094L)
stopifnot(all_converted_correlations$model$k == 19L)
stopifnot(sum(all_converted_correlations$data$N) == 8867L)

# ---------------------------------------------------------------------------
# 6. LEAVE-ONE-OUT ANALYSIS
# ---------------------------------------------------------------------------

leave_one_out <- do.call(
  rbind,
  lapply(seq_len(nrow(primary)), function(index) {
    selected <- primary[-index, ]
    fitted_model <- rma(
      yi = yi,
      vi = vi,
      data = selected,
      method = "REML",
      test = "z"
    )
    prediction <- predict(fitted_model, transf = transf.ztor)
    pooled_r <- as.numeric(transf.ztor(fitted_model$b))
    data.frame(
      Omitted_study = primary$Study[index],
      k = fitted_model$k,
      Total_N = sum(selected$N),
      Pooled_r = pooled_r,
      CI_lower = as.numeric(transf.ztor(fitted_model$ci.lb)),
      CI_upper = as.numeric(transf.ztor(fitted_model$ci.ub)),
      p_value = as.numeric(fitted_model$pval),
      Q = as.numeric(fitted_model$QE),
      Q_p = as.numeric(fitted_model$QEp),
      I2_percent = as.numeric(fitted_model$I2),
```

```r
      tau2 = as.numeric(fitted_model$tau2),
      PI_lower = as.numeric(prediction$pi.lb),
      PI_upper = as.numeric(prediction$pi.ub),
      Direction_changed = pooled_r >= 0,
      Significance_changed = fitted_model$pval >= 0.05,
      stringsAsFactors = FALSE
    )
  })
)

leave_one_out_ranges <- data.frame(
  Metric = c("Pooled r", "I2 (%)", "tau2"),
  Minimum = c(
    min(leave_one_out$Pooled_r),
    min(leave_one_out$I2_percent),
    min(leave_one_out$tau2)
  ),
  Maximum = c(
    max(leave_one_out$Pooled_r),
    max(leave_one_out$I2_percent),
    max(leave_one_out$tau2)
  )
)

# ----------------------------------------------------------------------------
# 7. INFLUENCE DIAGNOSTICS
# ----------------------------------------------------------------------------

influence_results <- influence(model_primary)

# Obtain each diagnostic with its dedicated documented metafor function. This
# avoids relying on the internal component layout of influence() and is stable
# across metafor versions.
studentized_diagnostics <- as.data.frame(rstudent(model_primary))
hat_values <- as.numeric(hatvalues(model_primary))
Cook_values <- as.numeric(cooks.distance(model_primary))
DFBETAS_diagnostics <- as.data.frame(dfbetas(model_primary))

if (ncol(DFBETAS_diagnostics) < 1L) {
  stop("metafor::dfbetas() did not return a coefficient column.")
}

DFBETAS_intercept_values <- as.numeric(DFBETAS_diagnostics[[1]])

diagnostic_lengths <- c(
  rstudent = length(studentized_diagnostics$z),
  hat = length(hat_values),
  cook_distance = length(Cook_values),
  DFBETAS = length(DFBETAS_intercept_values)
)

if (any(diagnostic_lengths != nrow(primary))) {
  stop(
    "Influence diagnostics have unexpected lengths: ",
    paste(names(diagnostic_lengths), diagnostic_lengths, sep = " = ", collapse = "; ")
  )
}
```

```
influence_table <- data.frame(
  Study = primary$Study,
  r = primary$r_harmonized,
  Hat_value = hat_values,
  Deleted_studentized_residual = as.numeric(studentized_diagnostics$z),
  DFBETAS_intercept = DFBETAS_intercept_values,
  Cooks_distance = Cook_values,
  stringsAsFactors = FALSE
)

# Thresholds match the manuscript. They are diagnostic indicators and are not
# automatic criteria for excluding a study.
influence_table$Studentized_flag <-
  abs(influence_table$Deleted_studentized_residual) > 2
influence_table$Hat_flag <-
  influence_table$Hat_value > (2 / model_primary$k)
influence_table$Cook_flag <-
  influence_table$Cooks_distance > (4 / model_primary$k)
influence_table$DFBETAS_flag <-
  abs(influence_table$DFBETAS_intercept) > (2 / sqrt(model_primary$k))
influence_table$Any_flag <- apply(
  influence_table[, c(
    "Studentized_flag", "Hat_flag", "Cook_flag", "DFBETAS_flag"
  )],
  1,
  any
)

# ---------------------------------------------------------------------------
# 8. PUBLICATION-BIAS / SMALL-STUDY-EFFECT ASSESSMENT
# ---------------------------------------------------------------------------

Egger_test <- regtest(
  model_primary,
  model = "lm",
  predictor = "sei",
  ret.fit = TRUE
)

Egger_coefficients <- coef(Egger_test$fit)
Egger_covariance <- vcov(Egger_test$fit)

if (length(Egger_coefficients) < 2L || nrow(Egger_covariance) < 2L) {
  stop("The fitted Egger regression did not return the predictor coefficient.")
}

# In metafor's weighted yi ~ SE parameterization, coefficient 2 is algebraically
# equivalent to the classical Egger intercept from the standardized-normal-
# deviate versus precision formulation.
Egger_intercept <- as.numeric(Egger_coefficients[2])
Egger_intercept_SE <- as.numeric(sqrt(diag(Egger_covariance))[2])

Egger_table <- data.frame(
  Test = "Egger regression test",
  Intercept = Egger_intercept,
  SE = Egger_intercept_SE,
```

```
  Statistic = as.numeric(Egger_test$zval),
  df = if (length(Egger_test$dfs)) as.numeric(Egger_test$dfs) else NA_real_,
  p_value = as.numeric(Egger_test$pval)
)

# ----------------------------------------------------------------------------
# 9. EXPLORATORY UNIVARIABLE META-REGRESSIONS
# ----------------------------------------------------------------------------

meta_regression_models <- list(
  Year_centered_at_2023 = rma(
    yi,
    vi,
    mods = ~ Year_centered_2023,
    data = primary,
    method = "REML",
    test = "z"
  ),
  MISSCARE_vs_other = rma(
    yi,
    vi,
    mods = ~ MISSCARE_binary,
    data = primary,
    method = "REML",
    test = "z"
  ),
  PES_NWI_vs_other = rma(
    yi,
    vi,
    mods = ~ PES_NWI_binary,
    data = primary,
    method = "REML",
    test = "z"
  )
)

meta_regression_reference <- c(
  Year_centered_at_2023 = "Year centered at 2023",
  MISSCARE_vs_other = "Other missed-care instrument",
  PES_NWI_vs_other = "Other practice-environment measure"
)

extract_meta_regression <- function(model_name, fitted_model) {
  data.frame(
    Moderator = model_name,
    Reference_or_centering = meta_regression_reference[[model_name]],
    Intercept = as.numeric(fitted_model$b[1]),
    Intercept_SE = as.numeric(fitted_model$se[1]),
    b = as.numeric(fitted_model$b[2]),
    SE = as.numeric(fitted_model$se[2]),
    CI_lower = as.numeric(fitted_model$ci.lb[2]),
    CI_upper = as.numeric(fitted_model$ci.ub[2]),
    p_value = as.numeric(fitted_model$pval[2]),
    QM = as.numeric(fitted_model$QM),
    QM_p = as.numeric(fitted_model$QMp),
    Residual_I2_percent = as.numeric(fitted_model$I2),
    Residual_tau2 = as.numeric(fitted_model$tau2),
```

```
    R2_percent = max(
      0,
      100 * (model_primary$tau2 - fitted_model$tau2) / model_primary$tau2
    ),
    stringsAsFactors = FALSE
  )
}

meta_regression_table <- do.call(
  rbind,
  Map(
    extract_meta_regression,
    names(meta_regression_models),
    meta_regression_models
  )
)

moderator_category_counts <- rbind(
  data.frame(
    Moderator = "MISSCARE_binary",
    Category = c("Other instrument", "MISSCARE family"),
    k = as.integer(table(factor(primary$MISSCARE_binary, levels = c(0, 1))))
  ),
  data.frame(
    Moderator = "PES_NWI_binary",
    Category = c("Other measure", "PES/NWI family"),
    k = as.integer(table(factor(primary$PES_NWI_binary, levels = c(0, 1))))
  )
)

# ---------------------------------------------------------------------------
# 10. REVIEWER-REQUESTED RISK-OF-BIAS SENSITIVITY ANALYSES
# ---------------------------------------------------------------------------

# The domains below reproduce the study-level JBI judgments reported in the
# Supplementary Materials. For analytical cross-sectional studies, the mapped
# key domains are exposure measurement (Q3), confounders identified (Q5),
# strategies for confounding (Q6), outcome measurement (Q7), and statistical
# analysis (Q8). For Lu et al. (2026), assessed with the JBI cohort checklist,
# the corresponding domains are Q3, Q4, Q5, Q7, and Q11. A rating of U means
# unclear; it is not silently treated as either Y or N.
risk_key_domains <- data.frame(
  Study = c(
    "Menard, 2014",
    "Kim et al., 2018",
    "Smith et al., 2018",
    "Ibrahim and Abohabieb, 2020",
    "Smith et al., 2020",
    "Elpasiony and Abd-Elmoghith, 2023",
    "Amiri Amjad et al., 2023",
    "Babaei et al., 2024",
    "Çiriş Yildiz et al., 2024",
    "Zhao et al., 2026",
    "Lu et al., 2026",
    "Kim and Uhm, 2026",
    "Campbell et al., 2020",
    "Liu et al., 2018",
```

```
    "El-Gazar et al., 2024"
  ),
  JBI_tool = c(
    rep("Analytical cross-sectional", 10),
    "Cohort",
    rep("Analytical cross-sectional", 4)
  ),
  Exposure_measurement = rep("Y", 15),
  Confounders_identified = c(
    "Y", "Y", "Y", "U", "N", "U", "U", "Y", "Y", "Y",
    "Y", "Y", "Y", "Y", "Y"
  ),
  Confounding_strategies = c(
    "Y", "Y", "Y", "U", "N", "U", "U", "Y", "Y", "Y",
    "Y", "Y", "Y", "Y", "Y"
  ),
  Outcome_measurement = rep("Y", 15),
  Statistical_analysis = rep("Y", 15),
  stringsAsFactors = FALSE
)

risk_match <- match(primary$Study, risk_key_domains$Study)
if (anyNA(risk_match) || anyDuplicated(risk_key_domains$Study)) {
  stop("Risk-of-bias classifications do not uniquely match all primary studies.")
}
risk_of_bias_primary <- risk_key_domains[risk_match, ]
stopifnot(identical(risk_of_bias_primary$Study, primary$Study))

risk_domain_columns <- c(
  "Exposure_measurement",
  "Confounders_identified",
  "Confounding_strategies",
  "Outcome_measurement",
  "Statistical_analysis"
)
risk_of_bias_primary$Explicit_N_in_key_domains <- apply(
  risk_of_bias_primary[, risk_domain_columns],
  1,
  function(values) any(values == "N")
)
risk_of_bias_primary$All_key_domains_yes <- apply(
  risk_of_bias_primary[, risk_domain_columns],
  1,
  function(values) all(values == "Y")
)

# Analysis A excludes only studies with an explicit N in at least one mapped
# key domain. Analysis B is deliberately stricter and retains only studies with
# Y in every mapped key domain, thereby excluding both N and U ratings.
risk_no_explicit_N <- fit_primary_subset(
  !risk_of_bias_primary$Explicit_N_in_key_domains,
  "Risk of bias: excluding any explicit N in mapped key domains"
)
risk_all_key_domains_yes <- fit_primary_subset(
  risk_of_bias_primary$All_key_domains_yes,
  "Risk of bias: all mapped key domains rated Y"
)
```

```
stopifnot(risk_no_explicit_N$model$k == 14L)
stopifnot(risk_all_key_domains_yes$model$k == 11L)

risk_of_bias_sensitivity_summary <- rbind(
  risk_no_explicit_N$summary,
  risk_all_key_domains_yes$summary
)
risk_of_bias_sensitivity_summary$Rule <- c(
  "Excluded studies with at least one explicit N; U ratings retained",
  "Retained only studies rated Y in all mapped key domains"
)
risk_of_bias_sensitivity_summary$Excluded_studies <- c(
  paste(
    risk_of_bias_primary$Study[
      risk_of_bias_primary$Explicit_N_in_key_domains
    ],
    collapse = "; "
  ),
  paste(
    risk_of_bias_primary$Study[
      !risk_of_bias_primary$All_key_domains_yes
    ],
    collapse = "; "
  )
)

# ---------------------------------------------------------------------------
# 11. REVIEWER-REQUESTED INSTRUMENT-COMPARABILITY SENSITIVITY ANALYSES
# ---------------------------------------------------------------------------

# These are restriction-based sensitivity analyses, not confirmatory subgroup
# comparisons. They show what happens when synthesis is limited to the most
# commonly used instrument families and when both constructs are measured with
# the two dominant families. The k = 6 intersection remains small and must be
# interpreted as exploratory.
instrument_MISSCARE <- fit_primary_subset(
  primary$MISSCARE_binary == 1,
  "Missed-care measure restricted to the MISSCARE family"
)
instrument_PES_NWI <- fit_primary_subset(
  primary$PES_NWI_binary == 1,
  "Work-environment measure restricted to the PES/NWI family"
)
instrument_both_families <- fit_primary_subset(
  primary$MISSCARE_binary == 1 & primary$PES_NWI_binary == 1,
  "Both MISSCARE-family and PES/NWI-family measures"
)

stopifnot(instrument_MISSCARE$model$k == 9L)
stopifnot(instrument_PES_NWI$model$k == 10L)
stopifnot(instrument_both_families$model$k == 6L)

instrument_sensitivity_summary <- rbind(
  instrument_MISSCARE$summary,
  instrument_PES_NWI$summary,
  instrument_both_families$summary
```

```
)
instrument_sensitivity_summary$Interpretation <- c(
  "Outcome-instrument restriction",
  "Exposure-instrument restriction",
  "Joint instrument-family restriction; exploratory because k = 6"
)

# ---------------------------------------------------------------------------
# 12. ADDITIONAL SMALL-STUDY-EFFECT SENSITIVITY ANALYSES
# ---------------------------------------------------------------------------

# The rank-correlation test and trim-and-fill analysis supplement, rather than
# replace, the Egger regression and visual funnel-plot assessment. With only 15
# studies and very high heterogeneity, all such analyses are exploratory.
rank_correlation_test <- ranktest(model_primary)
if (
  length(rank_correlation_test$tau) != 1L ||
  length(rank_correlation_test$pval) != 1L
) {
  stop("ranktest() did not return one Kendall tau estimate and one p-value.")
}

small_study_tests <- rbind(
  data.frame(
    Test = "Egger regression test",
    Estimate_type = "Regression coefficient for standard error",
    Estimate = Egger_intercept,
    SE = Egger_intercept_SE,
    Statistic = as.numeric(Egger_test$zval),
    df = if (length(Egger_test$dfs)) as.numeric(Egger_test$dfs) else NA_real_,
    p_value = as.numeric(Egger_test$pval),
    stringsAsFactors = FALSE
  ),
  data.frame(
    Test = "Begg-Mazumdar rank correlation test",
    Estimate_type = "Kendall tau",
    Estimate = as.numeric(rank_correlation_test$tau),
    SE = NA_real_,
    Statistic = NA_real_,
    df = NA_real_,
    p_value = as.numeric(rank_correlation_test$pval),
    stringsAsFactors = FALSE
  )
)

trimfill_model <- trimfill(
  model_primary,
  estimator = "L0",
  maxiter = 100
)
if (
  length(trimfill_model$k0) != 1L ||
  length(trimfill_model$side) != 1L
) {
  stop("trimfill() did not return one k0 estimate and one imputation side.")
}
trimfill_summary <- model_summary_r(
```

```
  "Exploratory Duval-Tweedie trim-and-fill sensitivity",
  trimfill_model,
  NA_real_
)
trimfill_summary$Observed_k <- model_primary$k
trimfill_summary$Estimated_missing_k <- as.integer(trimfill_model$k0)
trimfill_summary$Augmented_k <- trimfill_model$k
trimfill_summary$Imputation_side <- as.character(trimfill_model$side)
trimfill_summary$Estimator <- "L0"
trimfill_summary$Interpretation_note <- paste(
  "Exploratory sensitivity to one funnel-asymmetry mechanism;",
  "not a corrected primary estimate"
)

# ---------------------------------------------------------------------------
# 13. SEPARATE EXPLORATORY ODDS-RATIO META-ANALYSIS
# ---------------------------------------------------------------------------

odds_ratios$yi <- log(odds_ratios$OR)
odds_ratios$sei <- (
  log(odds_ratios$CI_upper) - log(odds_ratios$CI_lower)
) / (2 * qnorm(0.975))
odds_ratios$vi <- odds_ratios$sei^2

model_OR <- rma(
  yi = yi,
  vi = vi,
  data = odds_ratios,
  method = "REML",
  test = "z"
)

OR_prediction <- predict(model_OR, transf = exp)
OR_summary <- data.frame(
  Analysis = "Exploratory adjusted OR random-effects model",
  k = model_OR$k,
  Total_N = sum(odds_ratios$N),
  Pooled_OR = as.numeric(exp(model_OR$b)),
  CI_lower = as.numeric(exp(model_OR$ci.lb)),
  CI_upper = as.numeric(exp(model_OR$ci.ub)),
  p_value = as.numeric(model_OR$pval),
  Q = as.numeric(model_OR$QE),
  Q_df = model_OR$k - model_OR$p,
  Q_p = as.numeric(model_OR$QEp),
  I2_percent = as.numeric(model_OR$I2),
  tau2 = as.numeric(model_OR$tau2),
  PI_lower = as.numeric(OR_prediction$pi.lb),
  PI_upper = as.numeric(OR_prediction$pi.ub)
)

# ---------------------------------------------------------------------------
# 14. FIGURES
# ---------------------------------------------------------------------------

png(
  file.path(output_directory, "Figure_1_primary_forest.png"),
  width = 1800,
```

```
  height = 1500,
  res = 180
)
forest(
  model_primary,
  slab = primary$Study,
  atransf = transf.ztor,
  xlab = "Pearson r",
  refline = 0,
  header = c("Study", "r [95% CI]"),
  mlab = "REML pooled association"
)
dev.off()

png(
  file.path(output_directory, "Figure_2_without_Amiri_forest.png"),
  width = 1800,
  height = 1400,
  res = 180
)
forest(
  model_without_Amiri,
  slab = data_without_Amiri$Study,
  atransf = transf.ztor,
  xlab = "Correlation",
  refline = 0,
  header = c("Study", "r [95% CI]"),
  mlab = "Sensitivity model excluding Amiri Amjad"
)
dev.off()

png(
  file.path(output_directory, "Figure_3_OR_forest.png"),
  width = 1800,
  height = 1000,
  res = 180
)
forest(
  model_OR,
  slab = odds_ratios$Study,
  atransf = exp,
  xlab = "Odds ratio",
  refline = 1,
  header = c("Study", "OR [95% CI]"),
  mlab = "Exploratory REML pooled odds ratio"
)
dev.off()

png(
  file.path(output_directory, "Figure_4_primary_funnel.png"),
  width = 1400,
  height = 1200,
  res = 180
)
funnel(
  model_primary,
  yaxis = "sei",
```

```
  xlab = "Fisher z",
  ylab = "Standard error"
)
dev.off()

png(
  file.path(output_directory, "Figure_5_influence_plot.png"),
  width = 1800,
  height = 1600,
  res = 180
)
plot(influence_results)
dev.off()

png(
  file.path(output_directory, "Figure_6_Baujat_plot.png"),
  width = 1600,
  height = 1200,
  res = 180
)
baujat(model_primary)
dev.off()

png(
  file.path(output_directory, "Figure_7_trim_and_fill_funnel.png"),
  width = 1600,
  height = 1300,
  res = 180
)
funnel(
  trimfill_model,
  yaxis = "sei",
  xlab = "Fisher z",
  ylab = "Standard error",
  legend = list(show = "cis")
)
dev.off()

png(
  file.path(output_directory, "Figure_8_strict_risk_of_bias_forest.png"),
  width = 1800,
  height = 1350,
  res = 180
)
forest(
  risk_all_key_domains_yes$model,
  slab = risk_all_key_domains_yes$data$Study,
  atransf = transf.ztor,
  xlab = "Pearson r",
  refline = 0,
  header = c("Study", "r [95% CI]"),
  mlab = "All mapped JBI key domains rated Y"
)
dev.off()

png(
  file.path(output_directory, "Figure_9_joint_instrument_family_forest.png"),
```

```
  width = 1800,
  height = 1100,
  res = 180
)
forest(
  instrument_both_families$model,
  slab = instrument_both_families$data$Study,
  atransf = transf.ztor,
  xlab = "Pearson r",
  refline = 0,
  header = c("Study", "r [95% CI]"),
  mlab = "MISSCARE-family and PES/NWI-family restriction"
)
dev.off()

# ---------------------------------------------------------------------------
# 15. EXPORT ALL RESULTS
# ---------------------------------------------------------------------------

# REVIEWER REVISION: export a machine-readable audit trail of prespecified
# versus post-hoc analytical decisions.
analytic_decisions <- data.frame(
  Decision = c(
    "Eligibility criteria",
    "Primary total-score Pearson correlation model",
    "Random-effects REML estimation",
    "Effect-direction harmonization",
    "Hartung-Knapp adjustment",
    "Leave-one-out / influence diagnostics",
    "Amiri exclusion sensitivity",
    "Spearman / Kendall sensitivity analyses",
    "Risk-of-bias sensitivity analyses",
    "Instrument-family sensitivity analyses",
    "Exploratory meta-regressions",
    "Egger / rank-correlation / trim-and-fill"
  ),
  Status = c(
    rep("Prespecified before inspection of pooled results", 4),
    rep("Post hoc robustness/exploratory", 8)
  ),
  stringsAsFactors = FALSE
)

results_workbook <- list(
  ANALYTIC_DECISIONS = analytic_decisions,
  PRIMARY_SUMMARY = rbind(primary_summary, Hartung_Knapp_summary),
  TAU2_QPROFILE_CI = tau2_qprofile_table,
  SENSITIVITY = sensitivity_summary,
  OVERLAP_SENSITIVITY = overlap_sensitivity_summary,
  LEAVE_ONE_OUT = leave_one_out,
  LOO_RANGES = leave_one_out_ranges,
  INFLUENCE = influence_table,
  EGGER = Egger_table,
  SMALL_STUDY_TESTS = small_study_tests,
  TRIM_AND_FILL = trimfill_summary,
  META_REGRESSION = meta_regression_table,
  MODERATOR_COUNTS = moderator_category_counts,
```

```
  RISK_OF_BIAS_SENSITIVITY = risk_of_bias_sensitivity_summary,
  ROB_CLASSIFICATION = risk_of_bias_primary,
  INSTRUMENT_SENSITIVITY = instrument_sensitivity_summary,
  OR_SUMMARY = OR_summary,
  PRIMARY_DATA_USED = primary,
  ALL_CORRELATIONS_USED = all_correlations,
  OR_DATA_USED = odds_ratios
)

write.xlsx(
  results_workbook,
  file.path(output_directory, "COMPLETE_META_ANALYSIS_RESULTS_V5.xlsx"),
  overwrite = TRUE
)

write.csv(
  primary,
  file.path(output_directory, "PRIMARY_15_ANALYSIS_DATA.csv"),
  row.names = FALSE,
  fileEncoding = "UTF-8"
)
write.csv(
  all_correlations,
  file.path(output_directory, "ALL_CORRELATION_SENSITIVITY_DATA.csv"),
  row.names = FALSE,
  fileEncoding = "UTF-8"
)
write.csv(
  odds_ratios,
  file.path(output_directory, "OR_3_ANALYSIS_DATA.csv"),
  row.names = FALSE,
  fileEncoding = "UTF-8"
)

capture.output(
  sessionInfo(),
  file = file.path(output_directory, "sessionInfo.txt")
)

sink(file.path(output_directory, "console_results.txt"))
cat("PRIMARY REML MODEL\n")
print(model_primary)
cat("\nQ-PROFILE CONFIDENCE INTERVAL FOR TAU2 AND MODEL-BASED I2\n")
print(tau2_qprofile_CI)
cat("\nHARTUNG-KNAPP MODEL\n")
print(model_HK)
cat("\nCORRELATION SENSITIVITY ANALYSES\n")
print(sensitivity_summary)
cat("\nOVERLAP SENSITIVITY ANALYSES\n")
print(overlap_sensitivity_summary)
cat("\nLEAVE-ONE-OUT RESULTS\n")
print(leave_one_out)
cat("\nLEAVE-ONE-OUT RANGES\n")
print(leave_one_out_ranges)
cat("\nINFLUENCE DIAGNOSTICS\n")
print(influence_table)
cat("\nEGGER TEST\n")
```

```
print(Egger_test)
cat("\nMETA-REGRESSIONS\n")
invisible(lapply(meta_regression_models, print))
cat("\nMETA-REGRESSION SUMMARY TABLE\n")
print(meta_regression_table)
cat("\nMODERATOR CATEGORY COUNTS\n")
print(moderator_category_counts)
cat("\nRISK-OF-BIAS SENSITIVITY ANALYSES\n")
print(risk_of_bias_sensitivity_summary)
cat("\nSTUDY-LEVEL MAPPED JBI KEY DOMAINS\n")
print(risk_of_bias_primary)
cat("\nINSTRUMENT-COMPARABILITY SENSITIVITY ANALYSES\n")
print(instrument_sensitivity_summary)
cat("\nSMALL-STUDY EFFECT TESTS\n")
print(small_study_tests)
cat("\nBEGG-MAZUMDAR RANK-CORRELATION TEST\n")
print(rank_correlation_test)
cat("\nTRIM-AND-FILL SENSITIVITY ANALYSIS\n")
print(trimfill_model)
cat("\nEXPLORATORY ODDS-RATIO MODEL\n")
print(model_OR)
sink()

message(
  "Analysis complete. All results are saved in: ",
  normalizePath(output_directory)
)
```
