# Supplementary material for: The Association Between the Nursing Work Environment and Missed Nursing Care: A Systematic Review and Meta-Analysis
Source: Nurs Rep. 2026 Sep 15;16(9):336. doi: 10.3390/nursrep16090336 (PMC13610344; doi:10.3390/nursrep16090336)
Supplement: Supplementary file 1 [file nursrep-16-00336-s001.zip › File S3 PRISMA 2020 CHECKLIST.pdf]

# PRISMA 2020 Checklist

*Manuscript: The Association Between the Nursing Work Environment and Missed Nursing Care: A Systematic Review and Meta-Analysis*

| Section and Topic | Item # | Checklist item | Location where item is reported |
|---|---|---|---|
| **TITLE** | | | |
| Title | 1 | Identify the report as a systematic review. | Title page, p. 1. |
| **ABSTRACT** | | | |
| Abstract | 2 | See the PRISMA 2020 for Abstracts checklist. | Structured Abstract, p. 1. |
| **INTRODUCTION** | | | |
| Rationale | 3 | Describe the rationale for the review in the context of existing knowledge. | Introduction, pp. 1–2. |
| Objectives | 4 | Provide an explicit statement of the objective(s) or question(s) the review addresses. | Introduction and research question, pp. 2–3. |
| **METHODS** | | | |
| Eligibility criteria | 5 | Specify the inclusion and exclusion criteria for the review and how studies were grouped for the syntheses. | Methods, Sections 2.4 and 2.9, pp. 4 and 7–8. |
| Information sources | 6 | Specify all databases, registers, websites, organisations, reference lists and other sources searched or consulted to identify studies. Specify the date when each source was last searched or consulted. | Methods, Sections 2.3 and 2.5, pp. 3–5; Supplementary Table S1, pp. 1–2. |
| Search strategy | 7 | Present the full search strategies for all databases, registers and websites, including any filters and limits used. | Methods, Section 2.3, pp. 3–4; Supplementary Table S1, pp. 1–2. |
| Selection process | 8 | Specify the methods used to decide whether a study met the inclusion criteria of the review, including how many reviewers screened each record and each report retrieved, whether they worked independently, and if applicable, details of automation tools used in the process. | Methods, Section 2.5, pp. 4–5. |
| Data collection process | 9 | Specify the methods used to collect data from reports, including how many reviewers collected data from each report, whether they worked independently, any processes for obtaining or confirming data from study investigators, and if applicable, details of automation tools used in the process. | Methods, Section 2.6, p. 6. |
| Data items | 10a | List and define all outcomes for which data were sought. Specify whether all results that were compatible with each outcome domain in each study were sought (e.g. for all measures, time points, analyses), and if not, the methods used to decide which results to collect. | Methods, Sections 2.2, 2.4, and 2.6, pp. 3–4 and 6. |
| | 10b | List and define all other variables for which data were sought (e.g. participant and intervention characteristics, funding sources). Describe any assumptions made about any missing or unclear information. | Methods, Section 2.6, p. 6; Supplementary Table S3, pp. 3–6. |
| Study risk of bias assessment | 11 | Specify the methods used to assess risk of bias in the included studies, including details of the tool(s) used, how many reviewers assessed each study and whether they worked independently, and if applicable, details of automation tools used in the process. | Methods, Section 2.7, pp. 6–7; Supplementary Tables S4A–S4B, pp. 6–9. |
| Effect measures | 12 | Specify for each outcome the effect measure(s) (e.g. risk ratio, mean difference) used in the synthesis or presentation of results. | Methods, Section 2.8, p. 7. |

# PRISMA 2020 Checklist

| Section and Topic | Item # | Checklist item | Location where item is reported |
|---|---|---|---|
| Synthesis methods | 13a | Describe the processes used to decide which studies were eligible for each synthesis (e.g. tabulating the study intervention characteristics and comparing against the planned groups for each synthesis (item #5)). | Methods, Sections 2.4 and 2.9, pp. 4 and 7–8. |
| | 13b | Describe any methods required to prepare the data for presentation or synthesis, such as handling of missing summary statistics, or data conversions. | Methods, Sections 2.6, 2.8, and 2.9, pp. 6–8; Supplementary Dataset S1, p. 20. |
| | 13c | Describe any methods used to tabulate or visually display results of individual studies and syntheses. | Methods, Section 2.9, pp. 7–8; Tables 1–5, pp. 9–13; Supplementary Figures S1–S6, pp. 14–19. |
| | 13d | Describe any methods used to synthesize results and provide a rationale for the choice(s). If meta-analysis was performed, describe the model(s), method(s) to identify the presence and extent of statistical heterogeneity, and software package(s) used. | Methods, Section 2.9, pp. 7–8. |
| | 13e | Describe any methods used to explore possible causes of heterogeneity among study results (e.g. subgroup analysis, meta-regression). | Methods, Section 2.9, p. 8. |
| | 13f | Describe any sensitivity analyses conducted to assess robustness of the synthesized results. | Methods, Section 2.9, pp. 7–8. |
| Reporting bias assessment | 14 | Describe any methods used to assess risk of bias due to missing results in a synthesis (arising from reporting biases). | Methods, Section 2.9, p. 8. |
| Certainty assessment | 15 | Describe any methods used to assess certainty (or confidence) in the body of evidence for an outcome. | Not undertaken; no formal certainty-of-evidence assessment was performed. |
| **RESULTS** | | | |
| Study selection | 16a | Describe the results of the search and selection process, from the number of records identified in the search to the number of studies included in the review, ideally using a flow diagram. | Methods, Section 2.5, pp. 4–5; Results, Section 3.1, p. 8; Figure 1, p. 6; Supplementary Figure S5, p. 18. |
| | 16b | Cite studies that might appear to meet the inclusion criteria, but which were excluded, and explain why they were excluded. | Methods, Section 2.5, p. 5; Supplementary Tables S5–S6, pp. 8–9. |
| Study characteristics | 17 | Cite each included study and present its characteristics. | Results, Sections 3.2 and 3.9, pp. 8–10 and 13–14; Supplementary Table S3, pp. 3–6. |
| Risk of bias in studies | 18 | Present assessments of risk of bias for each included study. | Results, Section 3.3, p. 10; Supplementary Tables S4A–S4B, pp. 6–9. |

# PRISMA 2020 Checklist

| Section and Topic | Item # | Checklist item | Location where item is reported |
|---|---|---|---|
| Results of individual studies | 19 | For all outcomes, present, for each study: (a) summary statistics for each group (where appropriate) and (b) an effect estimate and its precision (e.g. confidence/credible interval), ideally using structured tables or plots. | Results, pp. 9–13; Supplementary Table S3, pp. 3–6; Supplementary Figures S1–S3, pp. 14–16; Supplementary Dataset S1, p. 20. |
| Results of syntheses | 20a | For each synthesis, briefly summarise the characteristics and risk of bias among contributing studies. | Results, Sections 3.2–3.4, pp. 8–10; Supplementary Tables S3–S4B, pp. 3–9. |
| | 20b | Present results of all statistical syntheses conducted. If meta-analysis was done, present for each the summary estimate and its precision (e.g. confidence/credible interval) and measures of statistical heterogeneity. If comparing groups, describe the direction of the effect. | Results, Sections 3.4–3.8, pp. 10–13; Tables 2–5, pp. 10–13; Supplementary Figures S1–S4, pp. 14–17. |
| | 20c | Present results of all investigations of possible causes of heterogeneity among study results. | Results, Section 3.7, pp. 12–13; Supplementary Table S2, p. 3. |
| | 20d | Present results of all sensitivity analyses conducted to assess the robustness of the synthesized results. | Results, Section 3.5, pp. 10–11; Table 3, p. 11; Supplementary Tables S7–S9, pp. 11–13; Supplementary Figures S2 and S6, pp. 15 and 19. |
| Reporting biases | 21 | Present assessments of risk of bias due to missing results (arising from reporting biases) for each synthesis assessed. | Results, Section 3.8, p. 13; Supplementary Figure S4, p. 17. |
| Certainty of evidence | 22 | Present assessments of certainty (or confidence) in the body of evidence for each outcome assessed. | Not undertaken; no formal certainty-of-evidence assessment was performed. |
| **DISCUSSION** | | | |
| Discussion | 23a | Provide a general interpretation of the results in the context of other evidence. | Discussion, Sections 4.1–4.4, pp. 14–16. |
| | 23b | Discuss any limitations of the evidence included in the review. | Discussion, Sections 4.3–4.4 and 4.7, pp. 15–18. |
| | 23c | Discuss any limitations of the review processes used. | Discussion, Section 4.7, pp. 17–18. |
| | 23d | Discuss implications of the results for practice, policy, and future research. | Discussion, Sections 4.5 and 4.8, pp. 16 and 18–19; Conclusions, p. 19. |

# PRISMA 2020 Checklist

| Section and Topic | Item # | Checklist item | Location where item is reported |
|---|---|---|---|
| **OTHER INFORMATION** | | | |
| Registration and protocol | 24a | The review was retrospectively registered in OSF on 25 August 2026 (DOI: 10.17605/OSF.IO/J62D3). No prospective protocol existed before review procedures began. | Methods 2.1: Retrospective OSF registration on 25 August 2026, DOI 10.17605/OSF.IO/J62D3; registration occurred after screening, extraction, and primary analysis. |
| | 24b | Indicate where the review protocol can be accessed, or state that a protocol was not prepared. | Methods 2.1: No prospective publicly available protocol existed before the review procedures began. |
| | 24c | Describe and explain any amendments to information provided at registration or in the protocol. | Methods 2.1 and 2.9: The manuscript distinguishes prespecified core decisions (eligibility criteria, primary total-score Pearson model, random-effects REML, direction harmonization) from post-hoc robustness/exploratory analyses. |
| Support | 25 | Describe sources of financial or non-financial support for the review, and the role of the funders or sponsors in the review. | Funding statement, p. 19. |
| Competing interests | 26 | Declare any competing interests of review authors. | Conflicts of Interest statement, p. 20. |
| Availability of data, code and other materials | 27 | Report which of the following are publicly available and where they can be found: template data collection forms; data extracted from included studies; data used for all analyses; analytic code; any other materials used in the review. | Supplementary Materials statement, pp. 19–20; Data Availability Statement, p. 20; Supplementary Dataset S1 and Files S1–S2. |

*From:* Page MJ, McKenzie JE, Bossuyt PM, Boutron I, Hoffmann TC, Mulrow CD, et al. The PRISMA 2020 statement: an updated guideline for reporting systematic reviews. BMJ 2021;372:n71. doi: 10.1136/bmj.n71. This work is licensed under CC BY 4.0. To view a copy of this license, visit https://creativecommons.org/licenses/by/4.0/
