# Supplementary material for: The Association Between the Nursing Work Environment and Missed Nursing Care: A Systematic Review and Meta-Analysis
Source: Nurs Rep. 2026 Sep 15;16(9):336. doi: 10.3390/nursrep16090336 (PMC13610344; doi:10.3390/nursrep16090336)
Supplement: Supplementary file 1 [file nursrep-16-00336-s001.zip › Supplementary Materials.pdf]

Supplementary Materials

The Association Between the Nursing Work Environment and Missed Nursing Care: A Systematic Review and Meta-Analysis

Final supplementary materials for the systematic review (40 included studies) and the primary random-effects meta-analysis of Pearson correlations (15 studies; 7,100 participants).

Analytic decision status and review timeline: Eligibility criteria, the primary total-score Pearson model, random-effects REML estimation, and direction harmonization were specified before inspection of pooled results. Hartung-Knapp, leave-one-out, Amiri exclusion, Spearman/Kendall, risk-of-bias and instrument-family sensitivity analyses, meta-regressions, influence diagnostics, and small-study-effect analyses were added post hoc. Initial search/screening: 15 March–15 June 2026; data extraction: 20 May–15 July 2026; final search update: 13 July 2026; statistical analysis: 16–22 July 2026; retrospective OSF registration: 25 August 2026.

Supplementary Table S1. Complete database-specific search strategies

Database/platform	Reproducible search documentation
PubMed	Final search date: 13 July 2026<br>Fields searched: Title/Abstract<br>Search limits and settings: No date, language, article-type, or full-text limits<br>Records retrieved: n = 44<br>Complete search string:<br>("nursing practice environment"[Title/Abstract] OR "nurse practice environment"[Title/Abstract] OR "professional practice environment"[Title/Abstract] OR "nursing work environment"[Title/Abstract] OR "practice environment scale"[Title/Abstract] OR "PES-NWI"[Title/Abstract]) AND ("missed nursing care"[Title/Abstract] OR "unfinished nursing care"[Title/Abstract] OR "care left undone"[Title/Abstract] OR "implicit rationing of nursing care"[Title/Abstract] OR "rationing of nursing care"[Title/Abstract] OR "omitted nursing care"[Title/Abstract] OR "nursing care omissions"[Title/Abstract]) AND (nurse[Title/Abstract] OR nurses[Title/Abstract] OR nursing[Title/Abstract])
Scopus	Final search date: 13 July 2026<br>Fields searched: TITLE-ABS-KEY<br>Search limits and settings: No date, language, document-type, or subject-area limits<br>Records retrieved: n = 55<br>Complete search string:<br>TITLE-ABS-KEY(("nursing practice environment" OR "nurse practice environment" OR "professional practice environment" OR "nursing work environment" OR "practice environment scale" OR "PES-NWI") AND ("missed nursing care" OR "unfinished nursing care" OR "care left undone" OR "implicit rationing of nursing care" OR "rationing of nursing care" OR "omitted nursing care" OR "nursing care omissions") AND nurs*)

Database/platform	Reproducible search documentation
Web of Science Core Collection	Final search date: 13 July 2026<br>Fields searched: TS (Topic)<br>Search limits and settings: No date, language, document-type, or Web of Science category limits<br>Records retrieved: n = 145<br>Complete search string:<br>TS=(("nursing practice environment" OR "nurse practice environment" OR "professional practice environment" OR "nursing work environment" OR "practice environment scale" OR "PES-NWI") AND ("missed nursing care" OR "unfinished nursing care" OR "care left undone" OR "implicit rationing of nursing care" OR "rationing of nursing care" OR "omitted nursing care" OR "nursing care omissions") AND nurs*)
APA PsycInfo via EBSCOhost	Final search date: 13 July 2026<br>Fields searched: TI (Title) and AB (Abstract)<br>Search limits and settings: Boolean/Phrase mode; no date, language, publication-type, peer-reviewed, or full-text limits; no expanders applied<br>Records retrieved: n = 48<br>Complete search string:<br>(TI ("nursing practice environment" OR "nurse practice environment" OR "professional practice environment" OR "nursing work environment" OR "practice environment scale" OR "PES-NWI") OR AB ("nursing practice environment" OR "nurse practice environment" OR "professional practice environment" OR "nursing work environment" OR "practice environment scale" OR "PES-NWI")) AND (TI ("missed nursing care" OR "unfinished nursing care" OR "care left undone" OR "implicit rationing of nursing care" OR "rationing of nursing care" OR "omitted nursing care" OR "nursing care omissions") OR AB ("missed nursing care" OR "unfinished nursing care" OR "care left undone" OR "implicit rationing of nursing care" OR "rationing of nursing care" OR "omitted nursing care" OR "nursing care omissions")) AND (TI nurs* OR AB nurs*)

Database/platform	Reproducible search documentation
Supplementary citation and targeted searching	Period: 15 March–15 June 2026; updated/finalized 13 July 2026<br>Sources: backward reference-list screening; forward citation tracking in Google Scholar and PubMed; publisher sites; exact-title searches; open-access repositories<br>Seed records: prior reviews by Chaboyer et al. and Zhao et al. (main references 10 and 11) and all provisionally eligible full-text reports<br>Core queries: "nursing practice environment" AND "missed nursing care"; "work environment" AND ("care left undone" OR "unfinished nursing care" OR "implicit rationing"); exact titles/DOIs of potentially eligible reports<br>Records retained after cross-source deduplication: n = 7

Note: The initial systematic search and screening process was conducted between 15 March and 15 June 2026. All database searches were rerun, updated, and finalized on 13 July 2026. No publication-date or language restrictions and no full-text or peer-reviewed filters were applied. APA PsycInfo was searched via EBSCOhost using TI and AB fields. The four finalized database searches retrieved 292 records before deduplication. CINAHL was unavailable through institutional access and was not searched. Supplementary citation tracking, reference-list screening, and targeted exact-title searches are documented in the final row.

Supplementary Table S2. Exploratory meta-regression analyses

Moderator	Reference / centering	Intercept	b	SE	95% CI	p	QM	Residual τ^2	R^2
Year of publication	Centered at 2023	−0.311	−0.0004	0.0175	−0.0346 to 0.0339	0.984	0.0004	0.053	0%
Missed-care instrument family (MISSCARE k = 9; other k = 6)	Other instrument	−0.380	0.118	0.120	−0.118 to 0.354	0.327	0.961	0.049	0%
Practice-environment instrument family (PES/NWI k = 10; other k = 5)	Other measure	−0.337	0.041	0.130	−0.213 to 0.295	0.753	0.099	0.053	0%

Note: b = meta-regression coefficient on the Fisher z scale; SE = standard error; CI = confidence interval; QM = omnibus moderator test; τ^2 = between-study variance; R^2 = estimated proportion of between-study heterogeneity explained. Each moderator was examined in a separate post-hoc univariable exploratory model (k = 15). Publication year was centered at 2023. The instrument-family comparisons were intentionally coarse; "other" categories contain conceptually heterogeneous instruments and the analyses are not tests of measurement equivalence.

Supplementary Table S3. Characteristics, extracted findings, synthesis category, and reason for non-inclusion in the primary Pearson meta-analysis

**Study**	**Country**	**Design / setting**	**N**	**Practice-environment measure**	**Missed-care measure**	**Main association / effect**	**Synthesis category**	Reason not included in primary Pearson meta-analysis
Menard, 2014	USA	Cross-sectional; acute care hospitals	557	PES-NWI / practice-environment measure	MISSCARE Survey	Pearson r = −0.510 after direction harmonization	Primary Pearson	Included in primary Pearson meta-analysis.
Papastavrou et al., 2014	Cyprus	Cross-sectional; hospital nurses	393	RPPE	BERNCA	Kendall τ = −0.234	Exploratory sensitivity	Reported Kendall τ rather than an eligible total-score Pearson r; exploratory converted-metric sensitivity only.
Hessels et al., 2015	USA	Cross-sectional; 70 hospitals	7,679	PES-NWI (hospital level)	Missed nursing care	Standardized regression coefficient β = −0.666	Narrative/regression	Reported regression effect rather than an eligible total-score Pearson r.
Kim et al., 2018	South Korea	Cross-sectional; hospital nurses	186	PES-NWI	MISSCARE Survey	Pearson r = −0.430	Primary Pearson	Included in primary Pearson meta-analysis.
Park et al., 2018	USA	Multilevel cross-sectional; 1,583 units in 371 hospitals	31,650	Practice environment categories	Missed nursing care	OR = 0.377 (95% CI 0.335 to 0.425)	OR meta-analysis	Reported adjusted odds ratios for practice-environment categories; separate exploratory OR synthesis.
Smith et al., 2018	USA	Cross-sectional	233	PES-NWI composite	MISSCARE Survey	Pearson r = −0.200	Primary Pearson	Included in primary Pearson meta-analysis.
Ibrahim and Abohabieb, 2020	Egypt	Cross-sectional	136	Nursing work environment measure	Missed nursing care	Pearson r = −0.200; p = 0.02	Primary Pearson	Included in primary Pearson meta-analysis.

Study	Country	Design / setting	N	Practice-environment measure	Missed-care measure	Main association / effect	Synthesis category	Reason not included in primary Pearson meta-analysis
Lake et al., 2020 (labor and delivery)	USA	Cross-sectional; 247 hospitals	1,318	Nursing work environment categories	Missed nursing care	Adjusted OR = 0.38 (95% CI 0.29 to 0.51)	OR meta-analysis	Reported adjusted OR for work-environment categories; separate exploratory OR synthesis.
Lake et al., 2020 (panel study)	USA	Longitudinal panel; 458 hospitals	23,650 (2006); 14,935 (2016)	Practice environment	Missed nursing care	Improved environment associated with less missed care; panel effect reported	Narrative/longitudinal	Longitudinal/panel association; no eligible total-score Pearson r for primary pooling.
Smith et al., 2020	Australia	Cross-sectional; small rural hospitals	383	NWI-R:A total	Care left undone	Pearson r = −0.360	Primary Pearson	Included in primary Pearson meta-analysis.
Zeleníková et al., 2020	Czech Republic	Cross-sectional	513	RPPE total	MISSCARE total	Spearman ρ = −0.2141	Sensitivity analysis	Reported Spearman ρ rather than Pearson r; post-hoc correlation-metric sensitivity analysis.
Liu et al., 2021	China	Panel study; 108 units in 23 hospitals	1,582 (2014); 1,530 (2018); 108 units in 23 hospitals	Nursing work environment	Care left undone	Longitudinal association; exact total correlation not reported	Narrative/longitudinal	Longitudinal association without an eligible total-score Pearson r.
Bartoníčková et al., 2022	Czech Republic	Cross-sectional; pediatric nurses	138	PES-NWI	Missed nursing care	No significant overall association reported.	Narrative	No extractable eligible total-score Pearson coefficient.
Labrague, 2022	Philippines	Cross-sectional	624	Practice environment dimensions	Missed nursing care	Significant subscale-level regression coefficients	Narrative/regression	Reported dimension/subscale regression effects rather than eligible total-score Pearson r.

Study	Country	Design / setting	N	Practice-environment measure	Missed-care measure	Main association / effect	Synthesis category	Reason not included in primary Pearson meta-analysis
Paiva-Santos et al., 2022	Portugal	Cross-sectional	66	Nursing practice environment	Missed care / individualized care	Favorable-threshold wards reported less missed care	Narrative	Reported group/threshold association rather than eligible total-score Pearson r.
Simonetti et al., 2022	Chile	Cross-sectional; 45 hospitals	1,853	Hospital work environment	Missed care	Better work environment associated with less missed care	Narrative/regression	Reported regression/organizational associations rather than eligible total-score Pearson r.
Tamayo et al., 2022	Philippines	Cross-sectional; tertiary public hospital	147	PES-NWI	BERNCA / rationing	Overall environment B = −0.20; $p < 0.05$	Narrative/regression	Reported regression coefficient rather than eligible total-score Pearson r.
Zárate-Grajales et al., 2023	Mexico	Cross-sectional; 11 highly specialized hospitals	510	Work environment categories	Missed nursing care	Adjusted OR = 0.43 (95% CI 0.28 to 0.65)	OR meta-analysis	Reported adjusted OR from fractional regression; separate exploratory OR synthesis.
Amiri Amjad et al., 2023	Iran	Cross-sectional	380	PES-NWI overall	MISSCARE total	Pearson r = 0.285	Primary Pearson	Included in primary Pearson meta-analysis; positive coefficient retained as published.
Albsoul et al., 2023	Jordan	Cross-sectional; 10 hospitals	672	PES-NWI overall/subscales	MISSCARE Survey	Only subscale-to-total correlations; no overall total–total r	Narrative	Reported subscale-to-total correlations but no overall total-score-to-total-score Pearson r.
Elpasiony and Abd-Elmoghith, 2023	Egypt	Cross-sectional; medical and surgical departments	200	Nursing work environment	Missed nursing care	Pearson r = −0.229	Primary Pearson	Included in primary Pearson meta-analysis.
Babaei et al., 2024	Iran	Cross-sectional	255	NWI-PES overall	MISSCARE total	Pearson r = −0.180	Primary Pearson	Included in primary Pearson meta-analysis.

Study	Country	Design / setting	N	Practice-environment measure	Missed-care measure	Main association / effect	Synthesis category	Reason not included in primary Pearson meta-analysis
Bruyneel et al., 2024	Belgium	Cross-sectional; 75 ICUs	2,183	PES-NWI / staffing-resource environment	Unfinished nursing care	Work environment related to unfinished care; mediation analyses	Narrative/mediation	Mediation/regression evidence rather than eligible total-score Pearson r.
El-Gazar et al., 2024	Egypt	Cross-sectional; 5 hospitals	231	Nursing practice environment	Implicit rationing of care	Pearson r = −0.380	Primary Pearson	Included in primary Pearson meta-analysis.
Rivaz et al., 2024	Iran	Cross-sectional; SEM	300	Nursing Professional Practice Environment Questionnaire	MISSCARE Survey	SEM effects for environment dimensions	Narrative/SEM	Structural-equation-model effects rather than eligible total-score Pearson r.
Çiriş Yildiz et al., 2024	Türkiye	Cross-sectional; two city hospitals	433	Nursing work environment	Missed nursing care	Pearson r = −0.249	Primary Pearson	Included in primary Pearson meta-analysis.
Özmen and Arslan Yürimezoğlu, 2025	Türkiye	Cross-sectional; pediatric units	123	PES-NWI subscales	13 missed-care activities	Logistic regressions for individual activities; no total–total r	Narrative	Logistic regressions for individual missed-care activities; no eligible total-score Pearson r.
Viana Santana et al., 2025	Brazil	Cross-sectional; emergency room	102	Practice Environment Scale	MISSCARE-Brazil	No eligible overall effect estimate reported.	Narrative	No eligible overall total-score Pearson coefficient extracted.
Labrague et al., 2025	Oman	Cross-sectional / mediation	1,237	Nurse practice environment	Missed nursing care	Direct β = −0.2919; p = 0.015; indirect β = −0.055	Narrative/mediation	Mediation/regression effects rather than eligible total-score Pearson r.
Zhao et al., 2026	China	Cross-sectional / mediation	535	Nursing work environment scale	Missed Nursing Care Scale	Pearson r = −0.399	Primary Pearson	Included in primary Pearson meta-analysis.
Lu et al., 2026	China	Longitudinal; three time points	902	Clinical nursing work environment	Missed nursing care	Pearson r = −0.502 between T1 work environment and T3 missed nursing care	Primary Pearson	Included in primary Pearson meta-analysis.
Kim and Uhm, 2026	South Korea	Cross-sectional; general hospital	177	PES-NWI-5	Korean MISSCARE Survey	Pearson r = −0.510	Primary Pearson	Included in primary Pearson meta-analysis.

Study	Country	Design / setting	N	Practice-environment measure	Missed-care measure	Main association / effect	Synthesis category	Reason not included in primary Pearson meta-analysis
Giannarou et al., 2026	Greece	National cross-sectional; 28 public hospitals	676	PES-NWI	MISSCARE Survey	No total–total correlation reported; mixed RN/NA sample	Narrative	No overall total-score Pearson correlation; mixed RN/nursing-assistant sample.
Putri et al., 2022	Indonesia	Cross-sectional; hospital nurses	56	PES-NWI / multidimensional nursing work environment	MISSCARE / missed nursing care	Spearman ρ = 0.564; p < 0.001; direction harmonized for synthesis	Sensitivity analysis	Reported Spearman ρ rather than Pearson r; post-hoc correlation-metric sensitivity analysis.
Campbell et al., 2020	USA	Cross-sectional; hospital nurses	950	PES-NWI	PIRNCA	Pearson r = −0.423	Primary Pearson	Included in primary Pearson meta-analysis.
Liu et al., 2018	China	Cross-sectional; hospitals	1,542	PES-NWI	Nursing care left undone	Pearson r = −0.080	Primary Pearson	Included in primary Pearson meta-analysis.
Pragosa et al., 2026	Portugal	Repeated descriptive cross-sectional study; emergency services	390 (2018); 434 (2022)	PES-NWI	Care-related outcomes	No eligible total–total Pearson coefficient	Narrative	No eligible total-score Pearson coefficient.
Zeleníková et al., 2021	Czech Republic	Cross-sectional; 14 hospitals	805	PES-NWI total	PIRNCA total	Spearman ρ = −0.2502; possible partial overlap with the 2020 report could not be excluded	Spearman sensitivity	Reported Spearman ρ rather than Pearson r; post-hoc correlation-metric sensitivity analysis.
Caillet et al., 2025	France	Nationwide cross-sectional study; 61 ICUs	1,271	PES-NWI and two workload measures	Missed nursing care prevalence	No eligible overall total–total coefficient reported	Narrative	No eligible total-score Pearson coefficient for the primary model.
Caponnetto et al., 2025	Italy	National multicentre cross-sectional home-care study	3,949 nurses; 9,780 patients	PES-NWI staffing/resource and leadership subscales	MNC_HC	Subscale quantile-regression effects; no eligible overall total–total coefficient	Narrative/regression	Reported adjusted/subscale-level associations rather than eligible total-score Pearson r.

Note: N = sample size; CI = confidence interval; OR = odds ratio; r = Pearson correlation coefficient; ρ = Spearman correlation coefficient; τ = Kendall correlation coefficient; β = standardized regression coefficient; B = unstandardized regression coefficient; PES-NWI = Practice Environment Scale of the Nursing Work Index; RPPE = Revised Professional Practice Environment; BERNCA = Basel Extent of Rationing of Nursing Care; SEM = structural equation modeling; RN = registered nurse; NA = nursing assistant. The final column explicitly distinguishes systematic-review inclusion from eligibility for the strict primary total-score Pearson model.

Supplementary Table S4A. Detailed JBI appraisal matrix for analytical cross-sectional studies

Study	Q1	Q2	Q3	Q4	Q5	Q6	Q7	Q8
Menard, 2014	Y	Y	Y	NA	Y	Y	Y	Y
Papastavrou et al., 2014	Y	Y	Y	NA	Y	Y	Y	Y
Hessels et al., 2015	Y	Y	Y	NA	Y	Y	Y	Y
Kim et al., 2018	Y	Y	Y	NA	Y	Y	Y	Y
Park et al., 2018	Y	Y	Y	NA	Y	Y	Y	Y
Smith et al., 2018	Y	Y	Y	NA	Y	Y	Y	Y
Ibrahim and Abohabieb, 2020	Y	Y	Y	NA	U	U	Y	Y
Lake et al., 2020 (labor and delivery)	Y	Y	Y	NA	Y	Y	Y	Y
Smith et al., 2020	Y	Y	Y	NA	N	N	Y	Y
Zeleníková et al., 2020	Y	Y	Y	NA	N	N	Y	Y
Bartoníčková et al., 2022	Y	Y	Y	NA	N	N	Y	Y
Labrague, 2022	Y	Y	Y	NA	Y	Y	Y	Y
Paiva-Santos et al., 2022	Y	Y	Y	NA	N	N	Y	Y
Simonetti et al., 2022	Y	Y	Y	NA	Y	Y	Y	Y
Tamayo et al., 2022	Y	Y	Y	NA	Y	Y	Y	Y
Zárate-Grajales et al., 2023	Y	Y	Y	NA	Y	Y	Y	Y
Putri et al., 2022	Y	Y	Y	NA	N	N	Y	Y
Amiri Amjad et al., 2023	Y	Y	Y	NA	U	U	Y	Y
Albsoul et al., 2023	Y	Y	Y	NA	N	N	Y	Y
Elpasiony and Abd-Elmoghith, 2023	Y	Y	Y	NA	U	U	Y	Y
Babaei et al., 2024	Y	Y	Y	NA	Y	Y	Y	Y
Bruyneel et al., 2024	Y	Y	Y	NA	Y	Y	Y	Y
El-Gazar et al., 2024	Y	Y	Y	NA	Y	Y	Y	Y
Rivaz et al., 2024	Y	Y	Y	NA	Y	Y	Y	Y

Study	Q1	Q2	Q3	Q4	Q5	Q6	Q7	Q8
Çiriş Yildiz et al., 2024	Y	Y	Y	NA	Y	Y	Y	Y
Özmen and Arslan Yürimezoğlu, 2025	Y	Y	Y	NA	Y	Y	Y	Y
Viana Santana et al., 2025	Y	Y	Y	NA	U	U	Y	Y
Labrague et al., 2025	Y	Y	Y	NA	Y	Y	Y	Y
Zhao et al., 2026	Y	Y	Y	NA	Y	Y	Y	Y
Kim and Uhm, 2026	Y	Y	Y	NA	Y	Y	Y	Y
Giannarou et al., 2026	Y	Y	Y	NA	Y	Y	Y	Y
Campbell et al., 2020	Y	Y	Y	NA	Y	Y	Y	Y
Liu et al., 2018	Y	Y	Y	NA	Y	Y	Y	Y
Pragosa et al., 2026	Y	Y	Y	NA	U	U	Y	Y
Zeleníková et al., 2021	Y	Y	Y	NA	N	N	Y	Y
Caillet et al., 2025	Y	Y	Y	NA	Y	Y	Y	Y
Caponnetto et al., 2025	Y	Y	Y	NA	Y	Y	Y	Y

Note: Q1–Q8 correspond, in the official order, to the revised JBI analytical cross-sectional tool (Barker et al., 2026; main manuscript reference 13). Y = yes; N = no; U = unclear; NA = not applicable. Two reviewers independently completed item-level appraisal and resolved disagreements by consensus; no numerical quality score or exclusion threshold was used.

Supplementary Table S4B. Detailed JBI appraisal matrix for longitudinal/cohort studies

Study	Q1	Q2	Q3	Q4	Q5	Q6	Q7	Q8	Q9	Q10	Q11
Lake et al., 2020 (panel study)	Y	Y	Y	Y	Y	U	Y	Y	Y	Y	Y
Liu et al., 2021	Y	Y	Y	Y	Y	U	Y	Y	Y	Y	Y
Lu et al., 2026	NA	NA	Y	Y	Y	U	Y	Y	Y	Y	Y

Note: Q1–Q11 correspond, in the official order, to the revised JBI cohort tool (Barker et al., 2025; main manuscript reference 14). Y = yes; N = no; U = unclear; NA = not applicable. Two reviewers independently completed item-level appraisal and resolved disagreements by consensus; no numerical quality score or exclusion threshold was used.

Supplementary Table S5. Full-text reports excluded, with reasons

Full report citation	Principal reason for exclusion

Full report citation	Principal reason for exclusion
[14] Winsett RP, Rottet K, Schmitt A, Wathen E, Wilson D; Missed Nursing Care Collaborative Group. Medical surgical nurses describe missed nursing care tasks—Evaluating our work environment. Applied Nursing Research. 2016;32:128–133. doi:10.1016/j.apnr.2016.06.006.	Ineligible exposure: no separate validated overall multidimensional nursing practice-environment measure matching the review definition.
[15] Dutra CKR, Guirardello EB. Nurse work environment and its impact on reasons for missed care, safety climate, and job satisfaction: A cross-sectional study. Journal of Advanced Nursing. 2021;77(5):2398–2406. doi:10.1111/jan.14764.	Ineligible outcome: assessed reasons for missed care rather than the frequency or extent of missed nursing care required by the review.
[16] Campagna S, Conti A, Clari M, Basso I, Sciannameo V, Di Giulio P, Dimonte V. Factors Associated With Missed Nursing Care in Nursing Homes: A Multicentre Cross-sectional Study. International Journal of Health Policy and Management. 2022;11(8):1334–1341. doi:10.34172/ijhpm.2021.23.	Ineligible setting/exposure: nursing-home study without an eligible overall multidimensional nursing practice-environment predictor.
[17] Zúñiga F, Ausserhofer D, Hamers JPH, Engberg S, Simon M, Schwendimann R. Are Staffing, Work Environment, Work Stressors, and Rationing of Care Related to Care Workers' Perception of Quality of Care? A Cross-Sectional Study. Journal of the American Medical Directors Association. 2015;16(10):860–866. doi:10.1016/j.jamda.2015.04.012.	Ineligible population/setting: mixed care-worker sample in nursing homes; no separate eligible nurse-only effect for the review question.
[18] Khajoei R, et al. Missed nursing care and related factors: a cross-sectional study. BMC Nursing. 2025;24:375. doi:10.1186/s12912-025-02984-3.	Ineligible exposure: no standardized multidimensional nursing work/practice-environment measure meeting the review definition.
[19] Daraghmeh H, Ayed A, Salameh B, Fashafsheh I. Factors of Missed Nursing Care in Intensive Care Units: Palestinian Perspective. Critical Care Nursing Quarterly. 2024;47(1):62–70. doi:10.1097/CNQ.0000000000000494.	Ineligible exposure: examined missed-care frequency and reasons without an eligible multidimensional nursing practice-environment measure.

Note: Reports were excluded after full-text assessment when the exposure, outcome, setting, publication type, or available effect estimate did not meet the prespecified eligibility criteria. Each report is listed once under its principal reason for exclusion.

Supplementary Table S6. Reports sought but not retrieved

Report sought but not retrieved	Retrieval status	Eligibility impact
[20] Bagnasco A, Rossi S, Dasso N, Catania G, Zanini M, Aleo G, Scelsi S, Petralia P, Watson R, Sasso L. Exploring Care Left Undone in Pediatric Nursing. Journal of Patient Safety. 2022;18(6):e903–e911. doi:10.1097/PTS.0000000000001044.	Only secondary/summary material was available; the complete report was not retrieved.	Not assessed; eligibility could not be confirmed.

Note: These reports are not counted as full-text exclusions because the complete report was unavailable for a definitive eligibility decision.

Completed JBI risk-of-bias appraisal: method and interpretation

The appraisal used the revised JBI analytical cross-sectional tool and the revised JBI cohort tool, matched to study design. Two reviewers (M.M. and N.F.) independently completed the study-level assessments and resolved disagreements through discussion and consensus. No arbitrary numerical quality score or exclusion threshold was applied. JBI judgments were interpreted at domain level. For reviewer-requested post-hoc risk-of-bias sensitivity analyses, key domains

were mapped to exposure measurement, confounding identification/management, outcome measurement, and statistical analysis; unclear ratings were not silently treated as “Yes” or “No”.

Across the included studies, measurement and statistical-analysis criteria were generally adequate. The recurring concern was incomplete identification or control of confounding, especially in unadjusted cross-sectional analyses. Two reviewers independently completed the study-level JBI assessments and resolved disagreements by consensus.

Question key for Supplementary Table S4A (analytical cross-sectional studies)

Q1: Were the criteria for inclusion in the sample clearly defined?

Q2: Were the study subjects and the setting described in detail?

Q3: Was the exposure measured in a valid and reliable way?

Q4: Were objective, standard criteria used for measurement of the condition?

Q5: Were confounding factors identified?

Q6: Were strategies to deal with confounding factors stated?

Q7: Were the outcomes measured in a valid and reliable way?

Q8: Was appropriate statistical analysis used?

Question key for Supplementary Table S4B (cohort studies)

Q1: Were the compared groups similar and recruited from the same population?

Q2: Were exposures measured similarly to assign participants to exposed and unexposed groups?

Q3: Was the exposure measured in a valid and reliable way?

Q4: Were confounding factors identified?

Q5: Were strategies to deal with confounding factors stated?

Q6: Were participants free of the outcome at the start of the study or at the time of exposure?

Q7: Were the outcomes measured in a valid and reliable way?

Q8: Was the follow-up time reported and sufficient to allow outcomes to occur?

Q9: Was follow-up complete and, if not, were reasons for loss to follow-up described and explored?

Q10: Were strategies to address incomplete follow-up utilized?

Q11: Was appropriate statistical analysis used?

Methodological note: JBI appraisal was performed independently by two reviewers, with disagreements resolved by discussion and consensus. Item-level judgments are reported rather than a numerical quality score. The revised tools are cited in references 13 and 14 of the main manuscript.

Supplementary Table S7. Leave-one-out analysis of the primary Pearson meta-analysis

Omitted study	Pooled r	95% CI	I²	τ²	95% prediction interval	Conclusion changed
Menard, 2014	−0.284	−0.389 to −0.171	95.5%	0.048	−0.627 to 0.152	No
Kim et al., 2018	−0.291	−0.399 to −0.176	96.0%	0.051	−0.641 to 0.159	No
Smith et al., 2018	−0.307	−0.414 to −0.192	96.0%	0.052	−0.654 to 0.146	No
Ibrahim and Abohabieb, 2020	−0.307	−0.414 to −0.192	96.1%	0.052	−0.653 to 0.146	No
Smith et al., 2020	−0.296	−0.405 to −0.179	96.0%	0.053	−0.648 to 0.161	No
Elpasiony and Abd-Elmoghith, 2023	−0.305	−0.413 to −0.189	96.1%	0.053	−0.654 to 0.150	No
Amiri Amjad et al., 2023	−0.340	−0.413 to −0.263	91.3%	0.023	−0.581 to −0.045	No
Babaei et al., 2024	−0.309	−0.415 to −0.194	96.0%	0.052	−0.654 to 0.143	No
Çiriş Yildiz et al., 2024	−0.304	−0.412 to −0.188	96.0%	0.053	−0.654 to 0.152	No
Zhao et al., 2026	−0.293	−0.402 to −0.177	95.8%	0.052	−0.645 to 0.161	No
Lu et al., 2026	−0.284	−0.389 to −0.171	95.2%	0.048	−0.628 to 0.153	No
Kim and Uhm, 2026	−0.285	−0.390 to −0.172	95.8%	0.048	−0.629 to 0.153	No
Campbell et al., 2020	−0.291	−0.399 to −0.175	95.5%	0.052	−0.642 to 0.161	No
Liu et al., 2018	−0.316	−0.420 to −0.205	94.9%	0.049	−0.650 to 0.120	No
El-Gazar et al., 2024	−0.295	−0.403 to −0.178	96.1%	0.052	−0.647 to 0.160	No

Note: r = pooled Pearson correlation coefficient after omission of the named study; CI = confidence interval; I^2 = model-based percentage of total variability attributable to between-study heterogeneity, as returned by metafor; τ^2 = estimated between-study variance. Across all 15 models, the direction and statistical significance of the pooled association remained unchanged.

Supplementary Table S8. Influence diagnostics for the primary Pearson meta-analysis

Study	r	Hat value	Deleted studentized residual	DFBETAS	Cook's distance	Threshold assessment
Menard, 2014	−0.510	0.069	−1.175	−0.319	0.099	No threshold exceeded
Kim et al., 2018	−0.430	0.064	−0.649	−0.170	0.030	No threshold exceeded
Smith et al., 2018	−0.200	0.065	0.467	0.124	0.016	No threshold exceeded

Study	r	Hat value	Deleted studentized residual	DFBETAS	Cook's distance	Threshold assessment
Ibrahim and Abohabieb, 2020	−0.200	0.062	0.454	0.117	0.014	No threshold exceeded
Smith et al., 2020	−0.360	0.068	−0.293	−0.079	0.007	No threshold exceeded
Elpasiony and Abd-Elmoghith, 2023	−0.229	0.064	0.332	0.087	0.008	No threshold exceeded
Amiri Amjad et al., 2023	0.285	0.068	3.897	1.059	0.562	|deleted residual| > 2; Cook distance > 4/k; |DFBETAS| > 2/√k
Babaei et al., 2024	−0.180	0.066	0.562	0.149	0.023	No threshold exceeded
Çiriş Yildiz et al., 2024	−0.249	0.068	0.246	0.066	0.005	No threshold exceeded
Zhao et al., 2026	−0.399	0.069	−0.500	−0.136	0.019	No threshold exceeded
Lu et al., 2026	−0.502	0.070	−1.129	−0.309	0.093	No threshold exceeded
Kim and Uhm, 2026	−0.510	0.064	−1.122	−0.292	0.084	No threshold exceeded
Campbell et al., 2020	−0.423	0.070	−0.638	−0.175	0.032	No threshold exceeded
Liu et al., 2018	−0.080	0.070	1.075	0.296	0.086	No threshold exceeded
El-Gazar et al., 2024	−0.380	0.065	−0.390	−0.103	0.011	No threshold exceeded

Note: r = Pearson correlation coefficient; DFBETAS = standardized change in the pooled effect estimate after study deletion; k = number of studies. Values of |deleted studentized residual| > 2, Cook's distance > 4/k, hat value > 2/k, and |DFBETAS| > 2/√k were treated as signals warranting closer examination. Thresholds were diagnostic indicators and were not used as automatic exclusion rules. Amiri Amjad et al. was the only study exceeding the deleted-residual, Cook's-distance, and DFBETAS thresholds.

Supplementary statistical robustness results

The REML model with Hartung–Knapp adjustment yielded r = −0.301 (95% CI −0.411 to −0.182), $t(14) = -5.273$; $p < 0.001$. Q-profile 95% CIs were 0.025 to 0.125 for τ^2 and 91.8% to 98.2% for model-based I^2. Across leave-one-out models, pooled r ranged from −0.340 to −0.284 and model-based I^2 from 91.3% to 96.1%; no omission changed direction or statistical significance. Possible overlap between the two Zeleníková reports was addressed by omitting each separately from the Pearson-plus-Spearman model (Supplementary Table S9).

Supplementary Table S9. Sensitivity analysis for possible overlap between the Zeleníková 2020 and 2021 reports

Omitted report	k	Total N	Pooled r	95% CI	I²	τ²
Zeleníková et al., 2020	17	7,961	−0.311	−0.405 to −0.211	95.5%	0.047
Zeleníková et al., 2021	17	7,669	−0.309	−0.403 to −0.208	95.4%	0.048

Note: Each model included the 15 primary Pearson studies plus the remaining two Spearman studies after omission of the named Zeleníková report. CI = confidence interval; I² = model-based heterogeneity; τ² = between-study variance.

Supplementary Table S10. Study-level scoring direction and effect harmonization in the primary Pearson meta-analysis

Study	Work-environment measure	Missed-care measure	Analysis-ready handling	Sign reversed?	Reason
Menard, 2014	PES-NWI / practice-environment measure	MISSCARE Survey	Sign reversed for pooling; magnitude unchanged	Yes	Study-specific scoring orientation yielded the opposite algebraic direction from the review convention that negative = more favorable environment with less missed care.
Kim et al., 2018	PES-NWI	MISSCARE Survey	Retained as published	No	Already aligned with review convention.
Smith et al., 2018	PES-NWI composite	MISSCARE Survey	Retained as published	No	Already aligned with review convention.
Ibrahim and Abohabieb, 2020	Nursing work environment measure	Missed nursing care measure	Retained as published	No	Already aligned with review convention.
Smith et al., 2020	NWI-R:A	Care left undone	Retained as published	No	Already aligned with review convention.
Campbell et al., 2020	PES-NWI	PIRNCA	Retained as published	No	Already aligned with review convention.
Liu et al., 2018	PES-NWI	Nursing care left undone	Retained as published	No	Already aligned with review convention.
Elpasiony and Abd-Elmoghith, 2023	Nursing work environment measure	MISSCARE Survey	Retained as published	No	Already aligned with review convention.
Amiri Amjad et al., 2023	PES-NWI	MISSCARE Survey	Positive r = 0.285 retained as published	No	No verified scoring/reporting error justified reversal; directionally discrepant result was handled only in post-hoc sensitivity/influence analyses.

Study	Work-environment measure	Missed-care measure	Analysis-ready handling	Sign reversed?	Reason
Babaei et al., 2024	NWI-PES	MISSCARE Survey	Retained as published	No	Already aligned with review convention.
El-Gazar et al., 2024	PES-NWI	PIRNCA	Retained as published	No	Already aligned with review convention.
Çiriş Yildiz et al., 2024	Nursing work environment measure	Missed nursing care measure	Retained as published	No	Already aligned with review convention.
Zhao et al., 2026	Nursing Work Environment Scale	Missed Nursing Care Scale	Retained as published	No	Already aligned with review convention.
Lu et al., 2026	Clinical Nursing Work Environment Scale	Missed nursing care measure	Retained as published	No	Already aligned with review convention.
Kim and Uhm, 2026	PES-NWI-5	MISSCARE Survey	Retained as published	No	Already aligned with review convention.

Note: Direction harmonization was prespecified before inspection of pooled results. A sign was reversed only to align algebraic scoring direction with the review convention; coefficient magnitudes were not altered.

Supplementary Table S11. Post-hoc risk-of-bias sensitivity analyses

Rule	k	N	Pooled r	95% CI	I²	τ²	95% prediction interval	Excluded studies
Exclude any explicit N in mapped key domains	14	6,717	−0.296	−0.405 to −0.179	96.0%	0.0528	−0.648 to 0.161	Smith et al., 2020
Retain only studies rated Y in all mapped key domains	11	6,001	−0.358	−0.443 to −0.266	93.2%	0.0268	−0.611 to −0.038	Ibrahim & Abohabieb, 2020; Smith et al., 2020; Elpasiony & Abd-Elmoghith, 2023; Amiri Amjad et al., 2023

Note: These analyses were added post hoc in response to risk-of-bias concerns. Mapped key domains were exposure measurement, confounding identification/management, outcome measurement, and statistical analysis. U = unclear was retained in the first rule and excluded under the stricter all-Y rule.

Supplementary Table S12. Post-hoc instrument-family sensitivity analyses

Restriction	k	N	Pooled r	95% CI	p	I²	τ²	95% prediction interval
Missed-care measure restricted to MISSCARE family	9	2,557	−0.256	−0.409 to −0.089	0.003	94.7%	0.0656	−0.660 to 0.263
Work-environment measure restricted to PES/NWI family	10	4,894	−0.289	−0.431 to −0.132	<0.001	96.8%	0.0671	−0.681 to 0.232

Restriction	k	N	Pooled r	95% CI	p	I²	τ²	95% prediction interval
Both MISSCARE and PES/NWI families	6	1,788	−0.271	−0.495 to −0.014	0.039	96.7%	0.1049	−0.747 to 0.388

Note: These restriction-based analyses were added post hoc to examine measurement comparability. They preserved an inverse average association but did not resolve heterogeneity; the joint-family analysis is especially exploratory because k = 6.

Supplementary Table S13. Post-hoc assessment of funnel asymmetry and small-study effects

Method	Estimate	Statistic	p	Interpretation
Egger regression	SE coefficient = −0.705; SE = 3.880	t(13) = −0.182	0.859	No statistical evidence of funnel asymmetry.
Begg-Mazumdar rank correlation	Kendall τ = 0.105	—	0.627	No statistical evidence of rank-correlation asymmetry.
Duval-Tweedie trim-and-fill	Estimated missing studies = 0	—	—	No studies imputed; pooled estimate unchanged.

Note: All small-study-effect procedures are exploratory because only 15 primary studies were available and heterogeneity was very high. Non-significant results do not demonstrate absence of publication bias.

Supplementary Table S14. Comparability of studies in the post-hoc exploratory adjusted-odds-ratio synthesis

Study	Analysis N	Exposure	Reference	Outcome	Model	Adjustment variables	Adjusted OR [95% CI]
Park et al., 2018	1,463 in adjusted model	Good practice environment	Poor practice environment	Any missed nursing care	Multilevel logistic regression	Teaching status, hospital size, Magnet status, location, patient case mix, and unit type	0.377 [0.335, 0.425]
Lake et al., 2020 (labor and delivery)	1,318	Better work environment	Poor work environment	Any missed nursing care	Logistic regression accounting for clustering within hospitals	Nurse education (BSN+), RN experience, hospital bed size, technology status, teaching status	0.38 [0.29, 0.51]
Zárate-Grajales et al., 2023	510	Enhanced practice environment	Attenuated practice environment	Overall fractional missed-care index	Fractional multiple logistic regression / quasi-maximum likelihood	Age, sex, education, work experience, professional category, weekly workload, incoming/outgoing patients, patient load, shift, job satisfaction, teamwork, institution type, service	0.43 [0.28, 0.65]

Note: The **three** models differ in exposure categorization, outcome operationalization, analysis level, and covariate sets. Their pooled OR is therefore retained only as a post-hoc exploratory summary; individual adjusted ORs are emphasized for interpretation.

Supplementary Figure S1. Forest plot of the primary Pearson correlation meta-analysis

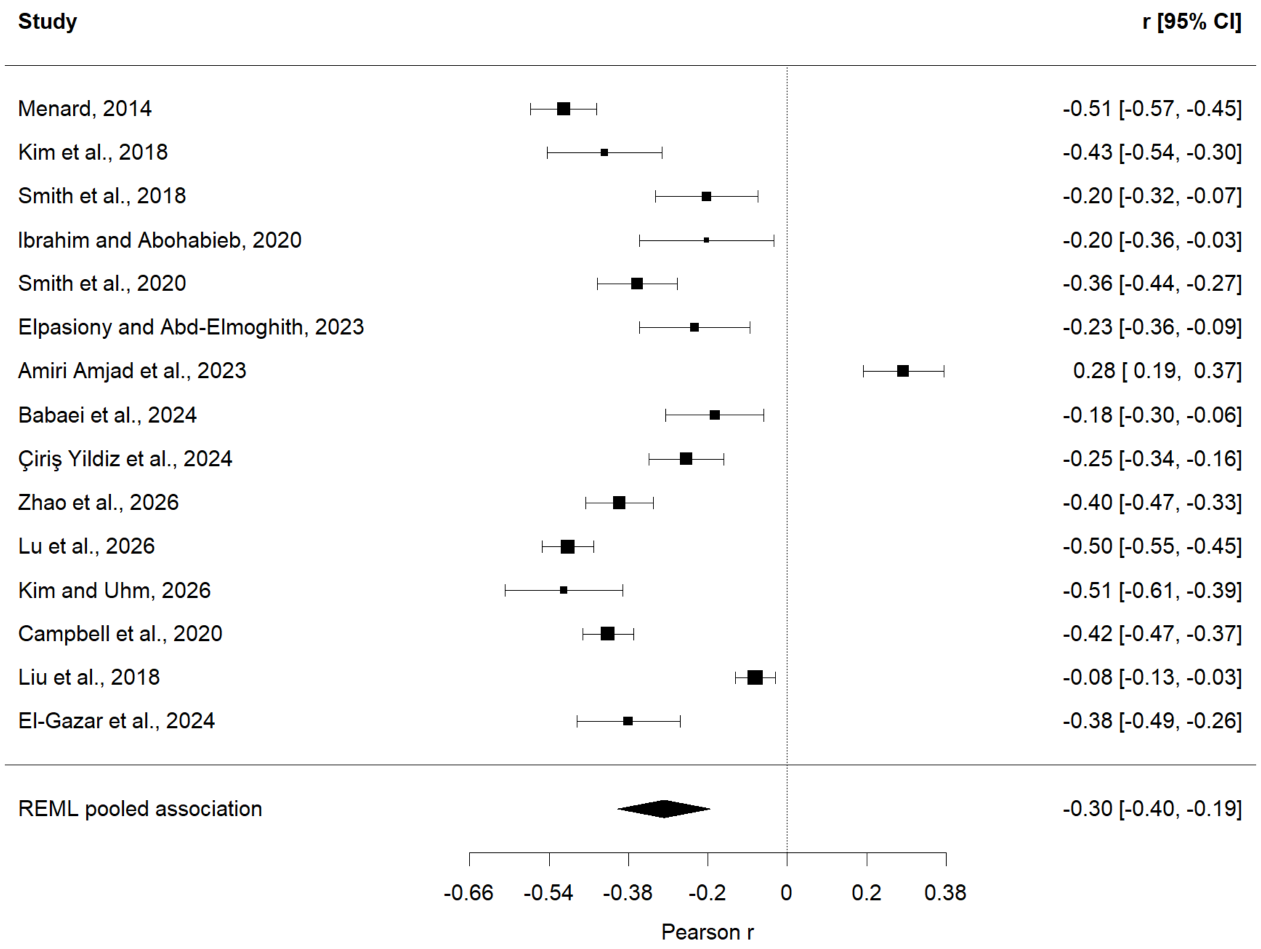

Random-effects REML model. Pooled r = −0.301; 95% CI −0.402 to −0.192; model-based I^2 = 95.7%; τ^2 = 0.049; 95% prediction interval −0.641 to 0.139.

Supplementary Figure S2. Forest plot of the sensitivity analysis excluding Amiri Amjad et al.

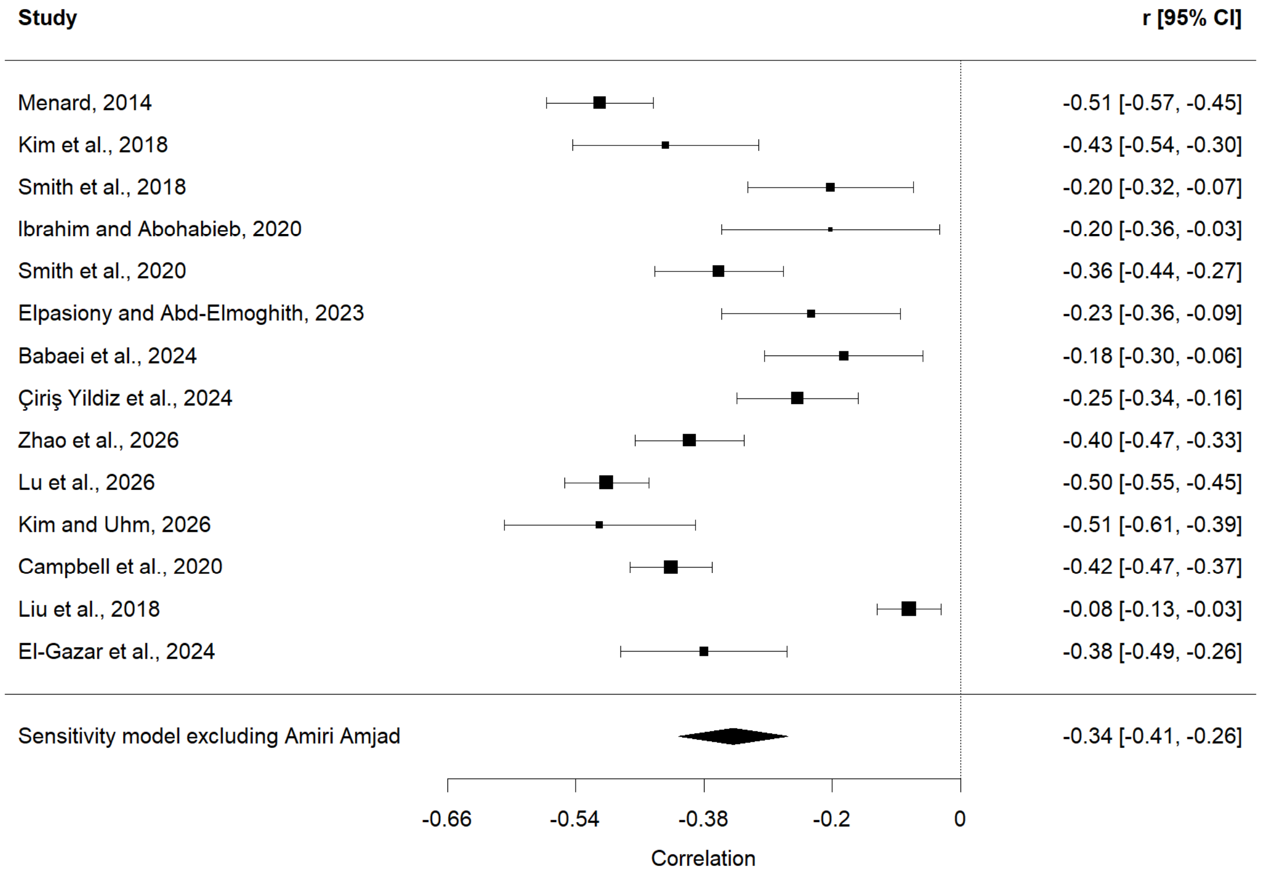

Random-effects REML sensitivity model. Pooled r = −0.340; 95% CI −0.413 to −0.263; model-based I^2 = 91.3%; τ^2 = 0.023; 95% prediction interval −0.581 to −0.045.

Supplementary Figure S3. Forest plot of the separate odds-ratio meta-analysis

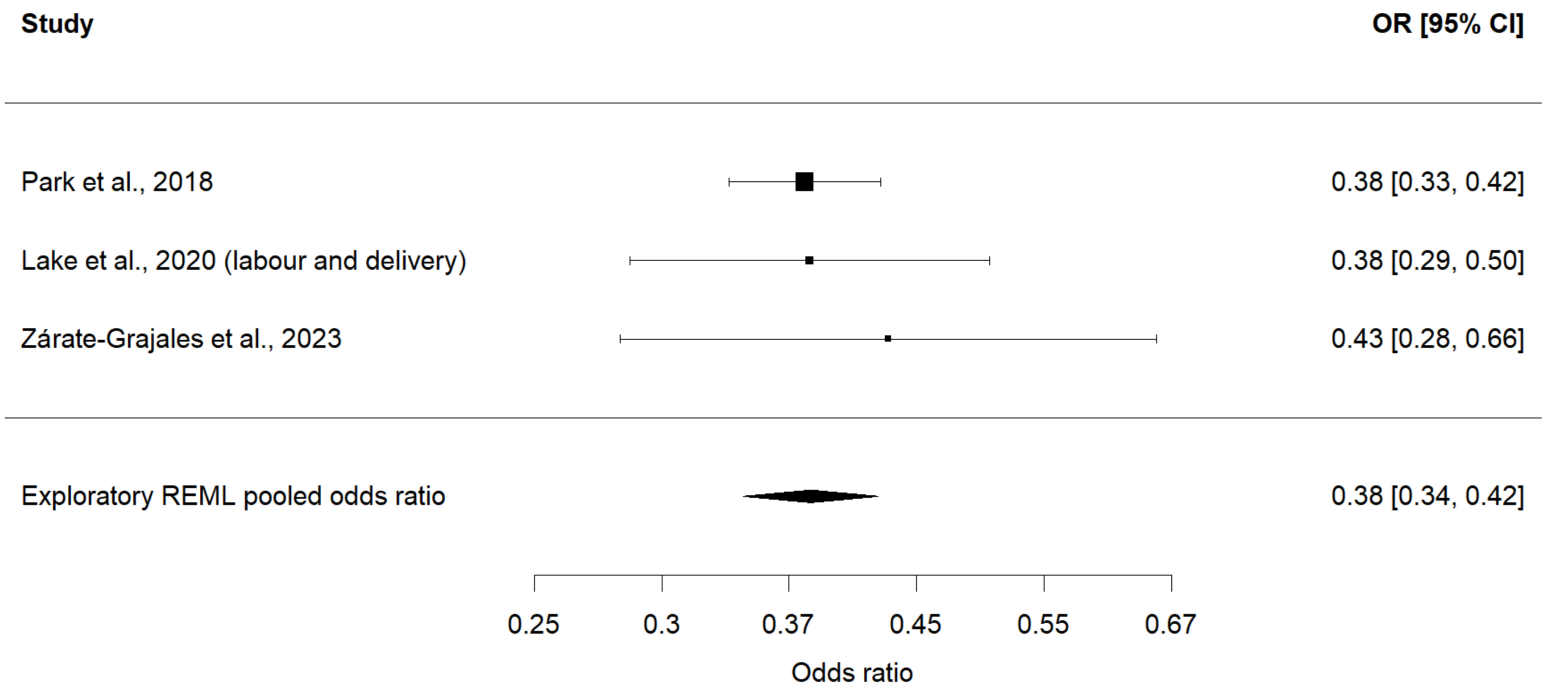

Exploratory random-effects REML model. Pooled OR = 0.381; 95% CI 0.342 to 0.423; I^2 = 0%. Individual study confidence intervals in the plot were reconstructed from log(OR) and its standard error and may differ by 0.01 from rounded published limits reported in Supplementary Table S3.

Supplementary Figure S4. Funnel plot for the primary Pearson correlation meta-analysis

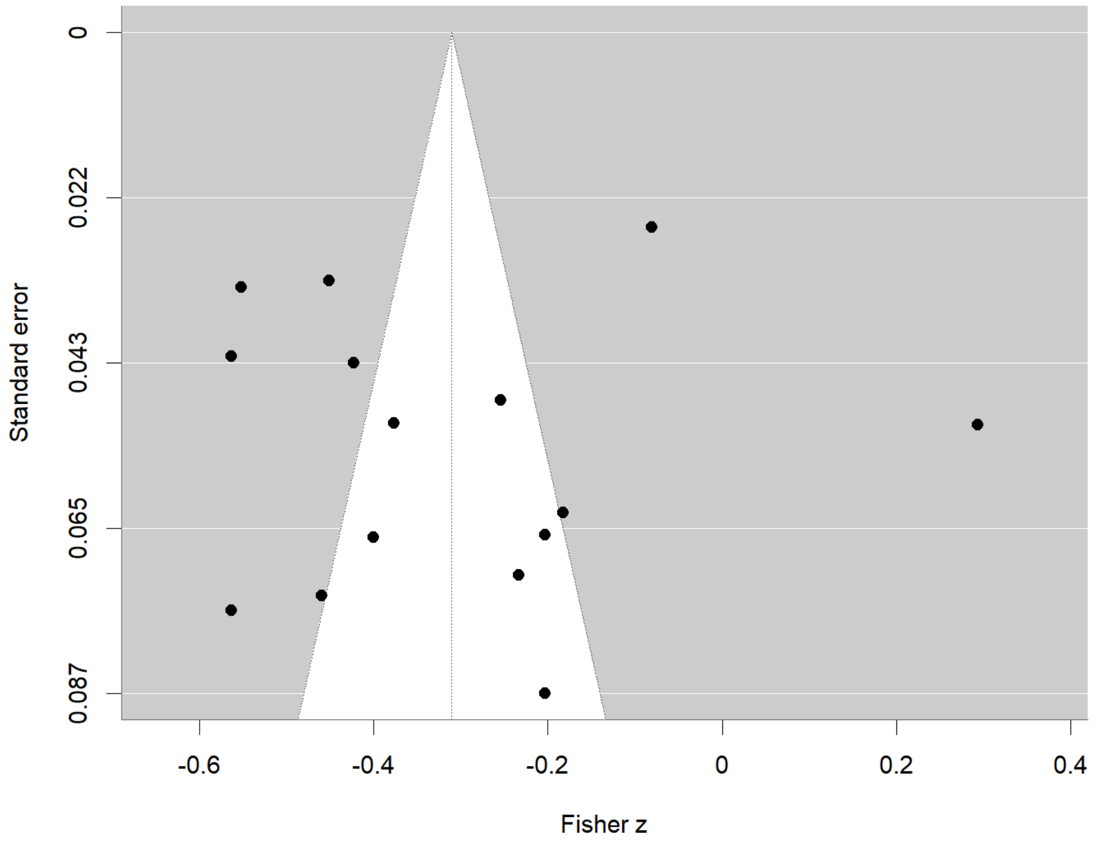

Egger regression coefficient for funnel-plot asymmetry = −0.705; SE = 3.879; t(13) = −0.182; p = 0.859. Interpretation is cautious because only 15 studies were included and heterogeneity was very high.

Supplementary Figure S5. Influence diagnostics for the primary Pearson meta-analysis

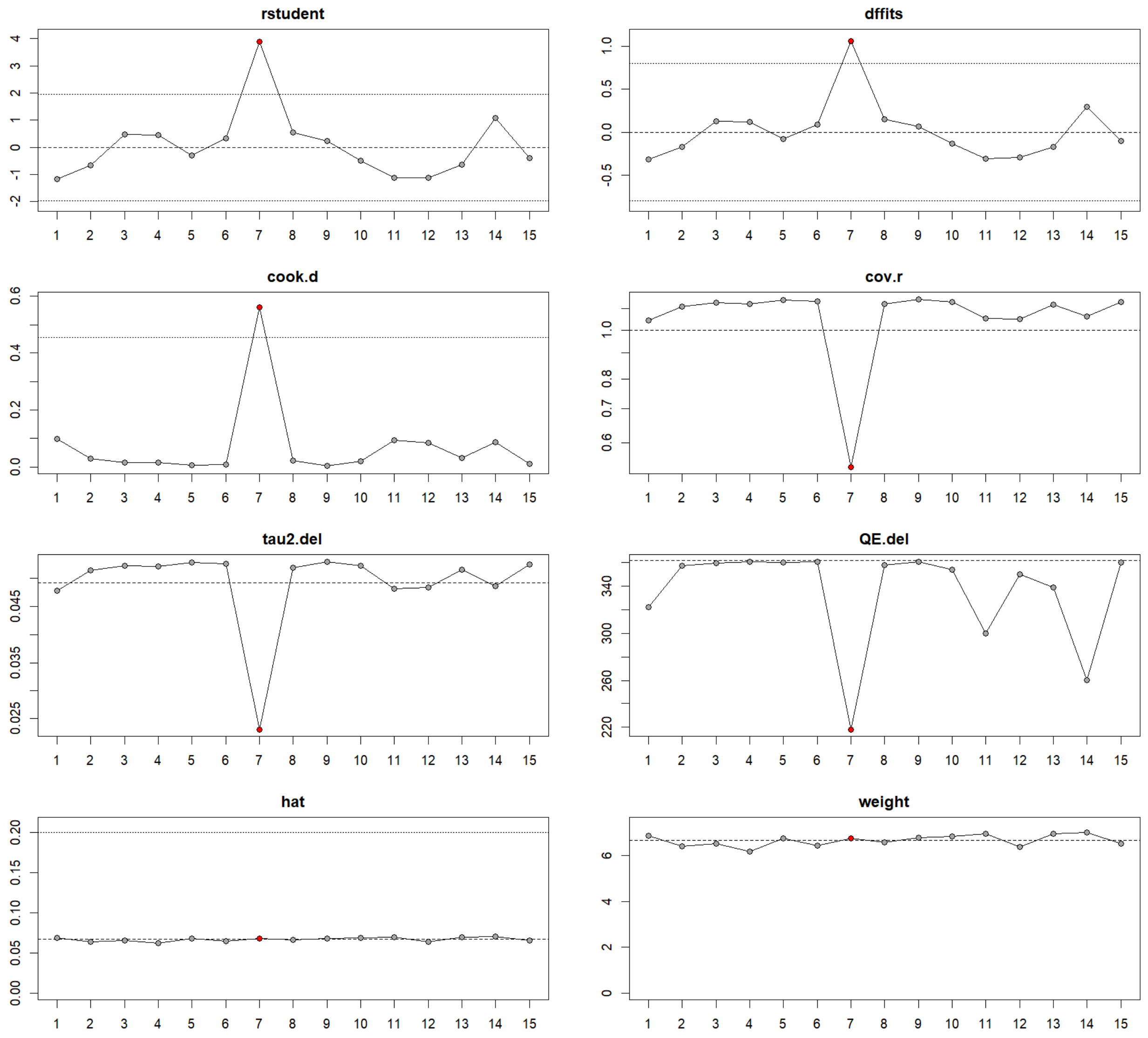

Study numbers follow the order in Supplementary Table S8 and Dataset S1; study 7 is Amiri Amjad et al. Red points indicate diagnostic threshold exceedances. Thresholds were used for examination, not as automatic exclusion criteria.

Supplementary Figure S6. Baujat plot for the primary Pearson meta-analysis.

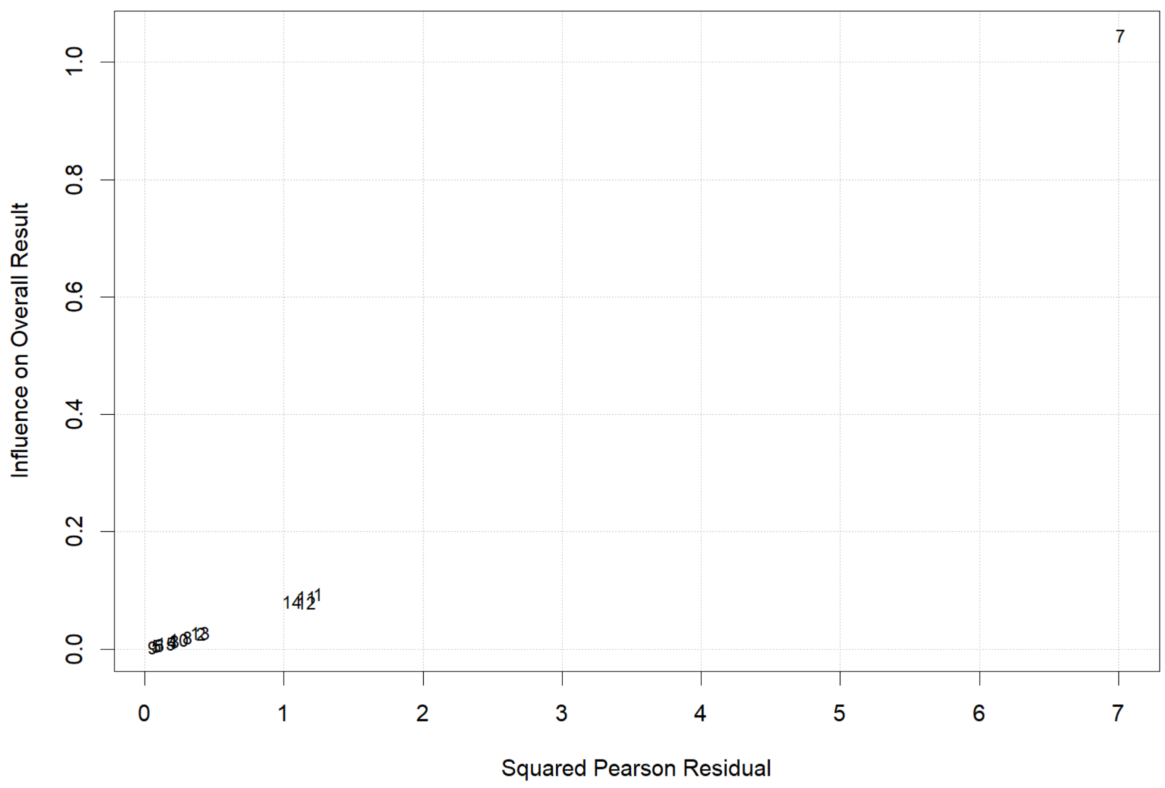

Supplementary Figure S7. Trim-and-fill funnel plot for the primary Pearson meta-analysis.

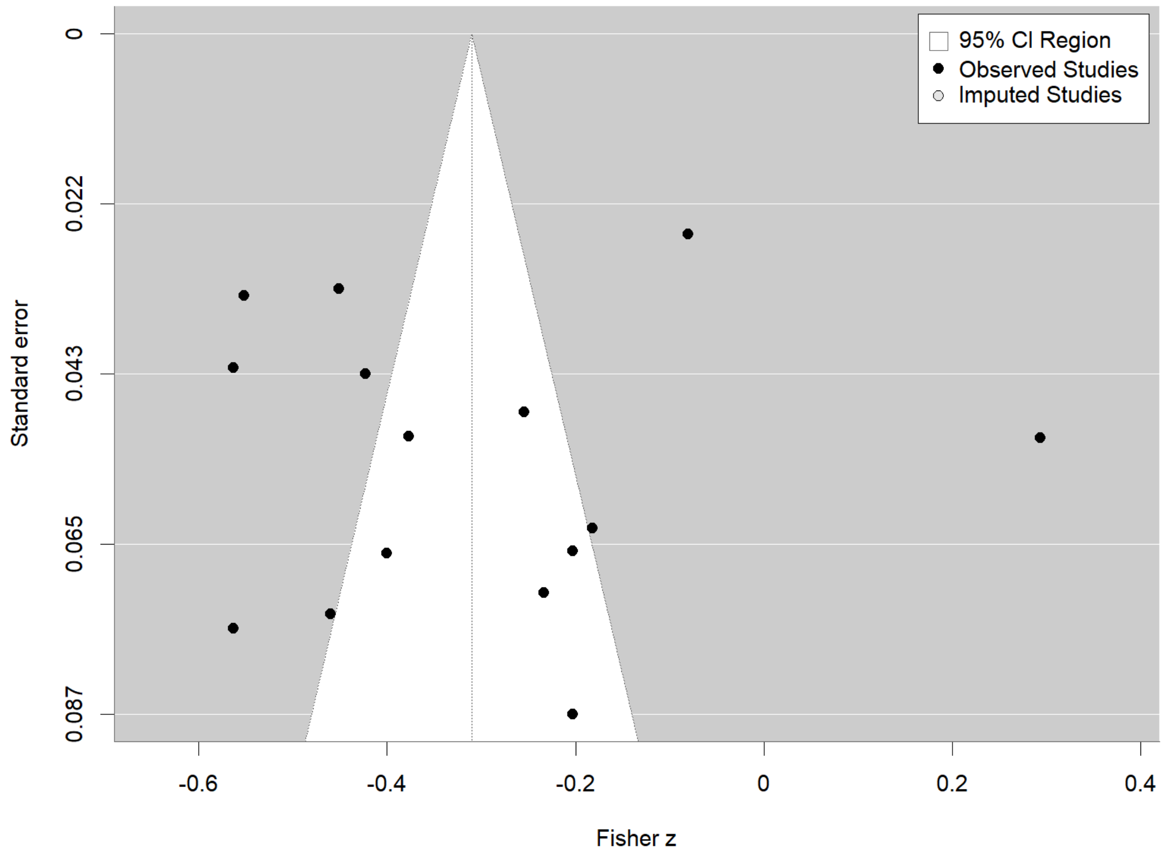

Note: Trim-and-fill estimated zero missing studies; this is an exploratory sensitivity analysis and not a corrected primary estimate.

Supplementary Figure S8. Forest plot of the strict risk-of-bias sensitivity analysis retaining only studies rated Y in all mapped key JBI domains.

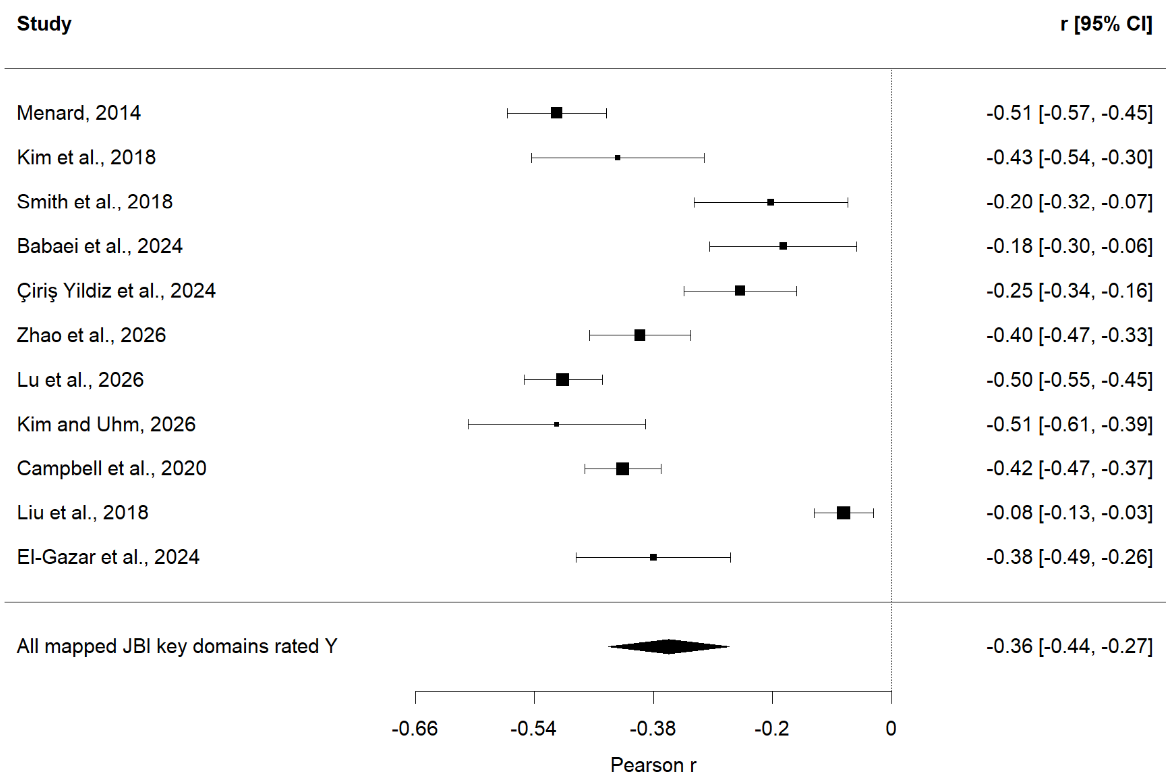

Pooled r = −0.358; 95% CI −0.443 to −0.266; I² = 93.2%; 95% prediction interval −0.611 to −0.038.

Supplementary Figure S9. Forest plot of the joint MISSCARE-family and PES/NWI-family instrument restriction.

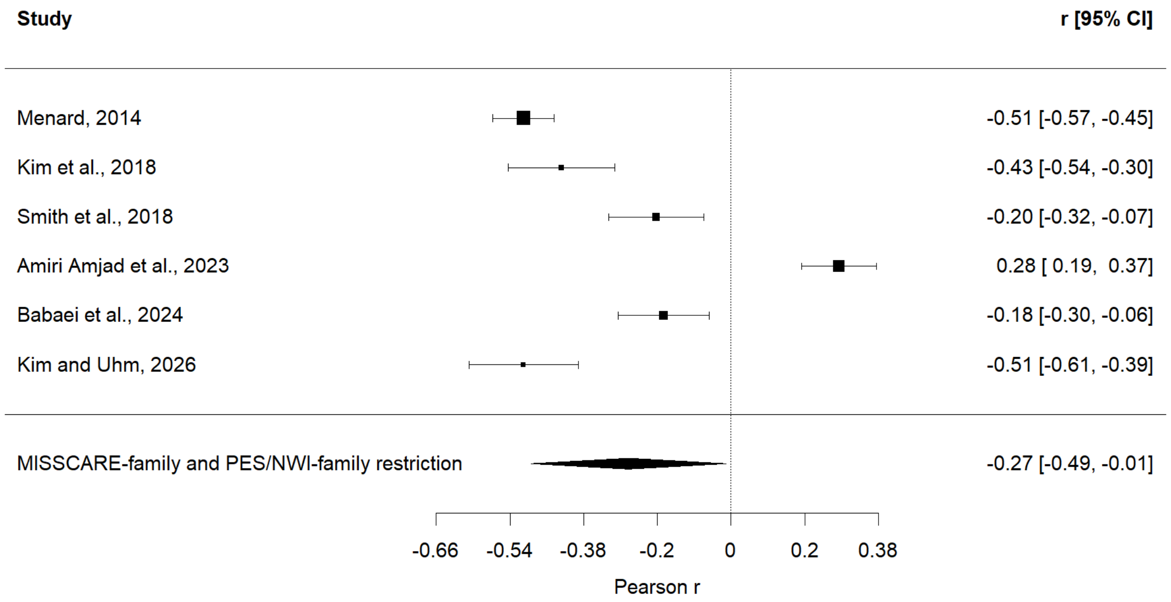

Pooled r = −0.271; 95% CI −0.495 to −0.014; I^2 = 96.7%. The joint restriction included only six studies and is exploratory.

Supplementary Dataset S1. Study-level meta-analysis dataset

Study	N	Effect measure	Effect used in synthesis	Scoring direction / harmonization	Fisher z or log(OR)	SE	Synthesis category
Menard, 2014	557	Pearson r	−0.510	Direction reversed to harmonize scoring	−0.562730	0.042486	Primary Pearson
Kim et al., 2018	186	Pearson r	−0.430	As published	−0.459897	0.073922	Primary Pearson
Smith et al., 2018	233	Pearson r	−0.200	As published	−0.202733	0.065938	Primary Pearson
Ibrahim and Abohabieb, 2020	136	Pearson r	−0.200	As published	−0.202733	0.086711	Primary Pearson
Smith et al., 2020	383	Pearson r	−0.360	As published	−0.376886	0.051299	Primary Pearson
Elpasiony and Abd-Elmoghith, 2023	200	Pearson r	−0.229	As published	−0.233134	0.071247	Primary Pearson
Amiri Amjad et al., 2023	380	Pearson r	0.285	Retained as published; positive direction flagged	0.293116	0.051503	Primary Pearson
Babaei et al., 2024	255	Pearson r	−0.180	As published	−0.181983	0.062994	Primary Pearson
Çiriş Yildiz et al., 2024	433	Pearson r	−0.249	As published	−0.254346	0.048224	Primary Pearson
Zhao et al., 2026	535	Pearson r	−0.399	As published	−0.422459	0.043355	Primary Pearson
Lu et al., 2026	902	Pearson r (T1–T3)	−0.502	T1 work environment; T3 missed care	−0.551976	0.033352	Primary Pearson
Kim and Uhm, 2026	177	Pearson r	−0.510	As published	−0.562730	0.075810	Primary Pearson
Campbell et al., 2020	950	Pearson r	−0.423	As published	−0.451340	0.032496	Primary Pearson
Liu et al., 2018	1,542	Pearson r	−0.080	As published	−0.080171	0.025491	Primary Pearson
El-Gazar et al., 2024	231	Pearson r	−0.380	As published	−0.400060	0.066227	Primary Pearson
Park et al., 2018	31,650	Adjusted OR	0.377	Better vs poor environment	−0.975510	0.060704	Separate OR meta-analysis
Lake et al., 2020 (labor and delivery)	1,318	Adjusted OR	0.380	Better vs poor; SE derived from published CI	−0.967584	0.144013	Separate OR meta-analysis
Zárate-Grajales et al., 2023	510	Adjusted OR	0.430	Enhanced vs attenuated environment	−0.843970	0.214843	Separate OR meta-analysis
Putri et al., 2022	56	Spearman ρ	−0.564	Direction reversed to harmonize scoring with the primary meta-analysis	−0.638680	0.137361	Spearman sensitivity
Zeleníková et al., 2020	513	Spearman ρ	−0.2141	As published	−0.217464	0.044281	Spearman sensitivity
Zeleníková et al., 2021	805	Spearman ρ	−0.2502	As published; possible partial sample overlap with 2020 report	−0.255626	0.035311	Spearman sensitivity
Papastavrou et al., 2014	393	Kendall τ	−0.359345	Converted from published τ = −0.234 using r = sin(π × τ / 2)	−0.376134	0.050637	Exploratory converted sensitivity

Note: For Pearson correlations, Fisher $z = 0.5 \times \ln[(1 + r)/(1 - r)]$ and $SE = \sqrt{1/(N - 3)}$. For odds ratios, ln(OR) was analyzed and SE was derived from the reported 95% confidence interval. Spearman coefficients were used only in sensitivity analyses. Kendall τ was converted using $r = \sin(\pi \times \tau / 2)$ for the exploratory analysis. The column "Effect used in synthesis" reports the analysis-ready value after any stated direction harmonization; it should not be interpreted as an unmodified published coefficient. For the OR studies, sample size is reported descriptively, whereas SE was calculated from the published confidence interval.
